# Dose reduction of biologics in patients with plaque psoriasis: a review

**DOI:** 10.3389/fphar.2024.1369805

**Published:** 2024-03-28

**Authors:** C. A. M. van Riel, C. A. J. Michielsens, M. E. van Muijen, L. S. van der Schoot, J. M. P. A. van den Reek, E. M. G. J. de Jong

**Affiliations:** ^1^ Radboud University Medical Centre, Nijmegen, Netherlands; ^2^ Department of Dermatology, Radboud University Medical Centre, Nijmegen, Gelderland, Netherlands; ^3^ Maastricht University Medical Centre, Maastricht, Limburg, Netherlands; ^4^ Department of Dermatology, Faculty of Health, Medicine and Life Sciences, Maastricht University, Maastricht, Netherlands, Netherlands; ^5^ Radboud University, Nijmegen, Gelderland, Netherlands

**Keywords:** psoriasis, dose reduction, dose tapering, clinical practice, implementation, biologics, biologicals, (cost-)effectiveness

## Abstract

Dose reduction (DR) of first-generation biologics for plaque psoriasis (TNF-alpha inhibitors (i) and interleukin (IL)-12/23i) has been described in a previous scoping review. The literature on the DR of the newest generation of biologics (IL-17/23i) was scarce. The current review provides a literature update on the previous scoping review on the DR of all biologics, including the newest generation, with a focus on the uptake and implementation of DR in practice. The current literature search on DR revealed 14 new articles in addition to those in the previous review. Four of the newly found articles tested DR strategies, mostly focusing on first-generation biologics; only guselkumab (IL-23i) was included in one study. The other 10 studies showed data on regaining response after failure of DR, safety, cost-effectiveness, and uptake and implementation, as well as information about IL-17/23i. The eligibility criteria to start DR included both absolute and relative Psoriasis Area and Severity Index (PASI) scores (PASI ≤3/≤5/PASI 75–100) and/or Dermatology Life Quality Index (DLQI) ≤3/≤5, or BSA ≤1/≤2, or Physician Global Assessment (PGA) ≤1/0–2 during a period ranging from 12 weeks to ≥1 year. Most studies used PASI ≤5 and/or DLQI ≤5 or PGA ≤1 for ≥6 months. DR strategies were mostly performed by stepwise interval prolongation in two steps (to 67% of the standard dose, followed by 50%). Some studies of IL-17/23i reduced the dose to ±25%. The tested DR strategies on stepwise or fixed DR on TNF-αi and IL-12/23i (three studies), as well as one “on-demand” dosing study on IL-23i guselkumab, were successful. In the case of relapse of DR on TNF-αi and IL-12/23i, clinical effectiveness was regained by retreatment with the standard dose. All studies showed substantial cost savings with the biologic DR of TNF-αi and IL-12/23i. The identified barriers against the implementation of DR were mainly a lack of guidelines and scientific evidence on effectiveness and safety, and a lack of time and (technical) support. The identified facilitators were mainly clear guidelines, feasible protocols, adequate education of patients and physicians, and cost reduction. In conclusion, DR seems promising, but a research gap still exists in randomized, prospective studies testing DR strategies, especially of IL-17/23i, hampering the completion of guidelines on DR. Taking into account the identified barriers and facilitators most likely results in a more successful implementation of biologic DR in practice.

## 1 Introduction

Psoriasis is a chronic immune-mediated skin disease causing a global burden, both clinically and economically, and affects approximately 2%–3% of the world population (Ghoreschi et al., 2021). Treatment options for psoriasis have increased in the past decades with the introduction of biologics. The first generation of biologics consisted of the tumor necrosis factor-alpha (TNF-α) inhibitors (infliximab, adalimumab, etanercept, and certolizumab pegol) and the interleukin (IL-)12/-23 inhibitor (ustekinumab). The newest generation of biologics entered the market more recently and includes IL-17 inhibitors (secukinumab, ixekizumab, brodalumab, and bimekizumab) and IL-23 inhibitors (guselkumab, risankizumab, and tildrakizumab). Biologics have been proven to be effective in patients with moderate-to-severe plaque psoriasis (Armstrong et al., 2020). However, they are also expensive and carry a risk of adverse events like infections and injection site reactions (Scherer et al., 2010; Gisondi et al., 2015; Reich et al., 2015; Snast et al., 2017; Thomaidou and Ramot, 2019; Armstrong et al., 2020). In general, biologics are prescribed in standard dosages, although previous research showed that patients with good treatment responses might be overtreated with these standard dosages (Menting et al., 2015). Therefore, exploring possibilities for the dose reduction (DR) of biologics in patients with plaque psoriasis is important. DR by prolongation of the injection interval of adalimumab, etanercept, and ustekinumab has proven to be effective, safe, and cost-effective in patients with stable low disease activity (Atalay et al., 2020a). A previous scoping review by Michielsens et al. (2021) provided a broad overview of the available literature on DR in adult patients with plaque psoriasis up to April 2020. This review showed that the available literature regarding the DR of the newest generation of biologics was scarce. The availability of sufficient literature on DR of both the first- and newest generation of biologics, as well as on the implementation of DR strategies, is important for incorporating DR in clinical practice. Therefore, the aim of this review is to provide an update on the previous scoping review on biologic DR by Michielsens et al. (2021) of all biologics, including the newest generation biologics, with as new aspect the uptake and implementation of DR in clinical practice.

## 2 Methods

PubMed was searched for literature between 1 January 2020 and 5 July 2023. We chose 2020 as the search of the previous review by Michielsens et al. (2021) ended here (April 2020). The search strategy was based on the strategy of Michielsens et al.; terms on psoriasis, all available biologic therapies, and verbs associated with DR were added (Supplemental Appendix S1). Titles and abstracts were screened by two reviewers (CvR and JvdR), and one reviewer (CvR) assessed full articles on the inclusion and exclusion criteria. Discrepancies were resolved by a second reviewer (JvdR) and, if necessary, by a third reviewer (EdJ). All studies providing full-text original research data on the DR of biologics in adults with plaque psoriasis were included. The definition of DR included the administration of a lower dose per administration or injection interval prolongation. Prior to DR, the initial treatment had to be in accordance with the registered dose of the biologic. Some biologics have two registered doses (e.g., adalimumab); changing the higher registered dose to a lower registered dose was not considered DR. However, one exception was made regarding the IL-12/23 inhibitor ustekinumab since its doses are weight-dependent. Accordingly, if a patient with a weight >100 kg reduced the dose from 90 mg to 45 mg, this was considered DR. Data extraction was performed by CvR. To provide an overview of the total body of evidence on DR strategies, the predesigned charting form from the previous review (Michielsens et al., 2021) was complemented with data from the present review (Supplemental Appendix S2). This charting form included the following data: study characteristics, eligibility criteria for DR, strategy of DR, DR outcomes (% of patients with successful lower doses, Psoriasis Area and Severity Index [PASI], Physician Global Assessment [PGA], Dermatology Life Quality Index [DLQI], % of relapses, and % of flares), and retreatment strategy in the case of relapse after DR and its effectiveness. Data on safety, effect on the quality of life (QoL), costs, and implementation were also extracted when described. All the data were summarized narratively.

## 3 Results

The studies included in the previous review by Michielsens et al. are shown in detail in Supplemental Appendix S2. In summary, this previous review reported the results of 19 studies on the effectiveness of DR strategies of biologics for psoriasis, of which 14 studies investigated the DR of adalimumab, 9 of etanercept, 5 of infliximab, 8 of ustekinumab, 1 of secukinumab, and 1 of brodalumab (Michielsens et al., 2021). The definition of low disease activity as a measure of DR eligibility widely varied among the included studies, and DR strategies were also heterogeneous. Evidence of regaining response after relapse due to DR was scarce, but restored remission was shown. The studies did not show a significant effect of DR on the occurrence of safety issues. Some studies reported on cost savings, but a formal cost-effectiveness analysis could not be identified at that time (Michielsens et al., 2021).

### 3.1 Included new studies

**FIGURE 1:**
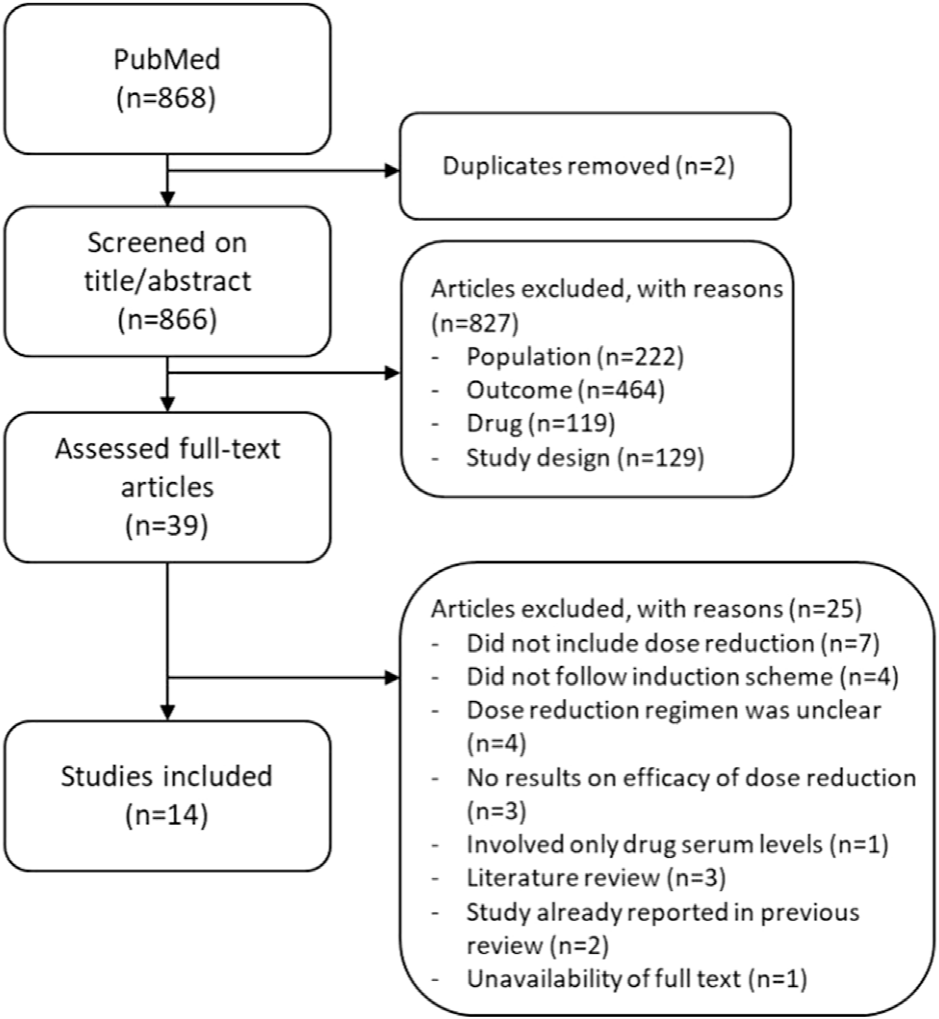
Flowchart of the study selection of 868 studies found between 1 January 2020 and 5 July 2023.

A total of 868 studies were screened for this updated review on title and abstract, of which 39 unique articles were selected for full-text screening. Eventually, 14 new articles were included (Figure 1). These articles involved four studies testing DR strategies (Atalay et al., 2021; Atalay et al., 2022b; Di Altobrando et al., 2022; Herranz-Pinto et al., 2023), one specifically focusing on the effectiveness of returning to standard dosages when DR failed (van der Schoot et al., 2022a), two addressing the safety of DR (Atalay et al., 2022a; Benzaquen et al., 2022), one evaluating the cost-effectiveness of DR (Atalay et al., 2020b), and six investigating the implementation and uptake of DR (Aubert et al., 2022; van der Schoot et al., 2022b; van Muijen et al., 2022; Aubert et al., 2023; van der Schoot et al., 2023a; van der Schoot et al., 2023b). All four studies testing DR strategies were cohort studies, of which three were prospective and one was retrospective. One study was a 1-year extension of a sub-cohort of the prospective CONDOR trial (Atalay et al., 2022b). The CONDOR trial is a multi-centric, randomized clinical trial (RCT) on the DR of adalimumab, etanercept, and ustekinumab, which was already highlighted in the previous scoping review by Michielsens et al. (Atalay et al., 2020a; Michielsens et al., 2021; Atalay et al., 2022b). The sub-cohort in the 1-year extension study comprised a total of 88 patients (single center) using either a reduced dose (N = 44/88) or standard dose (N = 44/88) of adalimumab, etanercept, or ustekinumab at the end of the CONDOR trial (Atalay et al., 2022b). The second study comprised a prospective observational cohort study, with a total of 80 patients using a one-step DR strategy of either adalimumab, etanercept, or ustekinumab in daily practice, who were observed for an average of 1 year (Atalay et al., 2021). The third prospective cohort study was by Di Altobrando et al. (2022), in which a total of 199 patients started a reduced dose (N = 96/199) or continued a standard dose (N = 103/199) of either adalimumab, etanercept, infliximab, or ustekinumab for maximal of ±102 months. Herranz-Pinto et al. (2023) performed a retrospective cohort study with a total of 69 patients, who started a reduced dose (N = 45/64) or continued the standard dose (N = 24/69) of guselkumab and were observed for a maximum of 90 weeks. Some studies were found in which a DR strategy of secukinumab, ixekizumab, brodalumab, and tildrakizumab was tested but were eventually excluded due to an uncertainty of which DR strategies were studied, whether an induction scheme was followed or not, or because DR was applied from the start of biologic use, or because results did not include effect measurements. The most frequently studied biologics were still first-generation biologics (adalimumab, etanercept, and ustekinumab). Six of the seven IL-17 and IL-23 inhibitors were mainly addressed in studies regarding costs, uptake, and implementation of DR and are described later.

### 3.2 Dose reduction strategies

#### 3.2.1 Eligibility criteria for dose reduction

The eligibility criteria used to start DR in the four pre-mentioned studies were roughly divided into two types: (i) the treatment duration of the biologic used in the standard dose prior to DR and (ii) the effectiveness of the biologic used in the standard dose at the moment of considering DR. In the 4 included studies, the treatment duration prior to DR ranged from ≥150 days (Herranz-Pinto et al., 2023) to ≥6 months (Atalay et al., 2021; Atalay et al., 2022b) to ≥1 year prior to DR (Di Altobrando et al., 2022). The effectiveness of the biologic used in the standard dose was determined by scoring the disease activity or state of clinical remission by using the absolute and/or relative PASI. The precise cut-off values of PASI varied between studies; however, all studies required low disease activity or a specific state of clinical remission for a certain period. Di Altobrando et al. (2022) chose a relative PASI 75–100 for ≥1 year, Herranz-Pinto et al. (2023) chose a complete response after 12 weeks (relative PASI 100), and both studies by Atalay et al. (2021) and Atalay et al. (2022b) used a PASI ≤5 for ≥6 months. Only the studies by Atalay et al. (2021); Atalay et al. (2022b) included the quality of life as an additional eligibility criterion, which was defined as a DLQI score of 5 or lower. The previous review by Michielsens et al. (2021) showed similar criteria regarding the treatment duration prior to DR and the effectiveness of the biologic used. The treatment duration prior to DR ranged from 6 weeks to ≥1 year, although the majority maintained a period of ≥ 6 months. In addition to the absolute and/or relative PASI score, the PGA or clinicians’ judgment was used to determine the effectiveness of the biologic used in the standard dose. Precise cut-off values also varied between studies, although all studies also required low disease activity or a certain state of clinical remission for a certain period of time ranging from a minimal of 6 weeks to ≥1 year. The CONDOR study was also the only study that used DLQI ≤ 5 as additional eligibility criteria (Atalay et al., 2020a). Only 2 of the 19 studies included in the previous review did not mention any eligibility criteria (Michielsens et al., 2021).

#### 3.2.2 Dose reduction strategies

In all four newly included studies, the induction phase of the biologics according to the standard dose prior to DR was followed. All studies applied DR by interval prolongation (Table 1). In the 1-year extension study by Atalay et al. (2022b), DR was performed stepwise by interval prolongation in two steps. The first step consisted of 67% of the standard dose (adalimumab every 3 weeks, etanercept every 10 days, and ustekinumab every 18 weeks), and the second step involved 50% of the standard dose (adalimumab every 4 weeks, etanercept every 2 weeks, and ustekinumab every 24 weeks) (Atalay et al., 2022b). In their other cohort study, DR was performed by fixed interval prolongation in one step: 67% of the standard dose (adalimumab every 3 weeks, etanercept every 10 days, and ustekinumab every 18 weeks) (Atalay et al., 2021). In the study by Di Altobrando et al. (2022), DR was also performed by fixed interval prolongation in one step but with different percentages of the standard dose ranging from 67% (adalimumab every 3 weeks and etanercept every 10 days) to 80% (infliximab every 10 weeks) to 86% (ustekinumab every 14 weeks). In the study by Herranz-Pinto et al. (2023), DR was performed by interval prolongation on-demand and showed that doses ranged from 73% (guselkumab every 11 weeks) to 47% (guselkumab every 17 weeks) to 30% (guselkumab every 27 weeks) of the standard dose. The studies included in the previous review applied DR by either interval prolongation or lowering the administration dose (Table 1) (Michielsens et al., 2021). However, Lebwohl et al. (2015) applied DR in both ways for brodalumab by increasing the interval in weeks while using 140 mg per administration instead of 210 mg. In addition, the study by van Bezooijen et al. (2017) was the only study that only lowered the administration dose by administering 45 mg of ustekinumab to a patient weighing >100 kg instead of 90 mg. All DR strategies used in the four included studies on DR strategy, complemented with the strategies used in the studies included in the previous review, are shown in Table 1. In summary, as shown in Table 1, the most frequently used strategies were either ±67% or ±50% of the standard dose of adalimumab, etanercept, ustekinumab, secukinumab, brodalumab, and guselkumab. Only studies involving infliximab did not go below 73% of the standard dose (Baniandres et al., 2015; Bardazzi et al., 2016; Romero-Jimenez et al., 2016; Di Altobrando et al., 2022). Only for brodalumab and guselkumab were lower DR strategies shown, i.e., 25% and 30% of the standard dose, respectively (Lebwohl et al., 2015; Herranz-Pinto et al., 2023). Similarly, Baniandres et al. (2015) applied a low DR of 33% and 35% of the standard dose in adalimumab and etanercept, respectively; however, this was done only in two patients for each biologic.

**TABLE 1:** Overview of all different dose reduction strategies used in the included studies testing dose reduction strategies by Michielsens et al. (2021) and the updated search. For each strategy, the references are shown as superscript.

Biologics		Standard dose	DR strategies	% of the standard dose
*First generation*				
**TNF-α inhibitor**	Adalimumab	40 mg Q2W	40 mg Q3W (Fotiadou et al., 2012; Lopez-Ferrer et al., 2013; Baniandres et al., 2015; Piaserico et al., 2016; Romero-Jimenez et al., 2016; Hansel et al., 2017; van Bezooijen et al., 2017; Atalay et al., 2020a; Atalay et al., 2021; Atalay et al., 2022b; Di Altobrando et al., 2022)	67%
			40 mg Q4W (Lopez-Ferrer et al., 2013; Taniguchi et al., 2013; Baniandres et al., 2015; Hansel et al., 2017; van Bezooijen et al., 2017; Lee et al., 2018; Atalay et al., 2020a; Atalay et al., 2022b)	50%
			40 mg Q6W (Baniandres et al., 2015)	33%
	Etanercept	50 mg QW	50 mg Q10D (Baniandres et al., 2015; Piaserico et al., 2016; Romero-Jimenez et al., 2016; Atalay et al., 2020a; Atalay et al., 2021; Atalay et al., 2022b; Di Altobrando et al., 2022)	70%
			50 mg Q14D (Baniandres et al., 2015; van Bezooijen et al., 2017; Atalay et al., 2020a; Atalay et al., 2022b)	50%
		25 mg 2x/W	25 mg QW (Baniandres et al., 2015)	50%
			25 mg Q10D (Baniandres et al., 2015)	35%
	Infliximab	5 mg/kg Q8W	5 mg/kg Q9W (Baniandres et al., 2015; Romero-Jimenez et al., 2016)	89%
			5 mg/kg Q10W (Bardazzi et al., 2016; Di Altobrando et al., 2022)	80%
			5 mg/kg Q11W (Baniandres et al., 2015)	73%
**IL-12/23 inhibitor**	Ustekinumab	Weight <100 kg 45 mg Q12W	45 mg Q13W (Baniandres et al., 2015; Romero-Jimenez et al., 2016)	92%
			45 mg Q14W (Baniandres et al., 2015; Di Altobrando et al., 2022)	86%
			45 mg Q16W (Blauvelt et al., 2017; van Bezooijen et al., 2017)	75%
			45 mg Q18W (Atalay et al., 2020a; Atalay et al., 2021; Atalay et al., 2022b)	67%
			45 mg Q20W (Blauvelt et al., 2017; van Bezooijen et al., 2017)	60%
			45 mg Q24W (Blauvelt et al., 2017; van Bezooijen et al., 2017; Atalay et al., 2020a; Atalay et al., 2022b)	50%
		Weight >100 kg 90 mg Q12W	90 mg Q16W (Blauvelt et al., 2017)	75%
			90 mg Q20W (Blauvelt et al., 2017)	60%
			90 mg Q24W (Blauvelt et al., 2017)	50%
			45 mg Q12W (van Bezooijen et al., 2017)	50% *
*Newest generation*				
**IL-17 inhibitor**	Secukinumab	300 mg Q4W	300 mg Q6W (Reich et al., 2020)	67%
	Brodalumab	210 mg Q2W	140 mg Q2W (Lebwohl et al., 2015)	67% *
			140 mg Q4W (Lebwohl et al., 2015)	50%
			140 mg Q8W (Lebwohl et al., 2015)	25%
**IL-23 inhibitor**	Guselkumab	100 mg Q8W	100 mg Q11W (Herranz-Pinto et al., 2023)	71%
			100 mg Q17W (Herranz-Pinto et al., 2023)	48%
			100 mg Q27W (Herranz-Pinto et al., 2023)	29%

DR, dose reduction; TNF, tumor necrosis factor; IL, interleukin; mg, milligram; Q, every; W, weeks; D, days; for example, Q2W meant every 2 weeks. * DR by lowering administration dose per administration.

### 3.3 Effectiveness of dose reduction

The effectiveness of the DR strategies was investigated in the four pre-mentioned studies (Atalay et al., 2021; Atalay et al., 2022b; Di Altobrando et al., 2022; Herranz-Pinto et al., 2023). Three of the four studies included adalimumab, etanercept, and ustekinumab, of which one study also included infliximab. One study included guselkumab. An overview of the results is given in Supplemental Appendix S2. One of the 14 included studies specifically focused on the effectiveness of *retreatment* in the case of relapse after the DR of adalimumab, etanercept, and ustekinumab. Detailed summaries on the design and outcomes regarding the effectiveness of DR are given in Supplemental Appendix S3.

#### 3.3.1 Atalay et al.—prospective cohort (N = 88) (1-year extension study of the randomized CONDOR trial) on adalimumab, etanercept, and ustekinumab

In the 1-year extension study of the CONDOR trial, a sub-cohort of a total of 88 patients was followed for another year after the end of the trial, resulting in a total follow-up of 2 years for this specific cohort (Atalay et al., 2022b). The sub-cohort comprised patients from one center who were initially randomized to a reduced dose (N = 44/88) or standard dose (usual care, UC) (N = 44/88) of adalimumab (DR N = 18; UC N = 17), etanercept (DR N = 11; UC N = 12), or ustekinumab (DR N = 15; UC N = 15) at the start of the CONDOR trial. The results were not specified per biologic but on a total study population level. At the end of the 1-year CONDOR trial, 59% of the patients initially randomized to DR (26/44 patients) were still on a low dose. At the end of the 1-year extension study (i.e., 2 years after CONDOR initiation), 69% of this group (18/26 patients) was still on a low dose (N = 7 used 67% of the standard dose and N = 11 used 50% of the standard dose). Over a total of 2 years of follow-up, 10 patients relapsed after DR, of which 80% (8/10 patients) regained response after retreatment with the previous effective dose (Atalay et al., 2022b).

#### 3.3.2 Atalay et al.—prospective cohort (N = 80) (one-step DR strategy) on adalimumab, etanercept, and ustekinumab

In this prospective cohort study, a total of 80 patients who started with a one-step DR strategy of adalimumab (N = 42), etanercept (N = 16), or ustekinumab (N = 22) were followed for, on average, 1 year after the start of DR (Atalay et al., 2021). DR was performed by fixed interval prolongation to 67% of the standard dose. Of the total study population, 45% (36/80 patients) discontinued DR (discontinuation of DR split per biologic: adalimumab, 45% (19/42 patients); etanercept, 44% (7/16 patients); and ustekinumab, 46% (10/22 patients)). Over the total follow-up period, a total of 8 out of 80 patients (10%) relapsed after DR, of which 50% (N = 4) continued DR at their own request and 50% (N = 4) returned to the standard dose. Response was regained within 6 months for 100% of patients who continued DR and 75% of patients who returned to the standard dose (3/4 patients) (Atalay et al., 2021).

#### 3.3.3 Di Altebrando et al.—prospective cohort (N = 199) on adalimumab, etanercept, infliximab, and ustekinumab

In this prospective cohort study, a total of 199 patients, of which 96 patients started with DR and 103 patients continued the standard dose (UC) of adalimumab (DR N = 47; UC N = 34), etanercept (DR N = 16; UC N = 25), infliximab (DR N = 21; UC N = 7), or ustekinumab (DR N = 12; UC N = 37), were followed for a maximum of ±102 months after the start of DR (Di Altobrando et al., 2022). The dose was reduced by fixed interval prolongation to 67% of the standard dose for adalimumab and etanercept, 80% for infliximab, and 86% for ustekinumab. During the follow-up, a total of 26 out of 96 patients (27%) on DR relapsed. For adalimumab DR, 36% (17/47 patients) relapsed; for etanercept DR, 6% (1/16 patients) relapsed; for infliximab DR, 24% (5/21 patients) relapsed; and for ustekinumab DR, 25% (3/12 patients) relapsed. Of all 26 relapsed patients, 96% (25/26 patients) regained their initial PASI score after retreatment with the standard dose (Di Altobrando et al., 2022).

#### 3.3.4 Herranz-Pinto et al.—retrospective cohort (N = 69) on guselkumab

This retrospective cohort study included a total of 69 patients, of which 45 underwent an “on-demand” DR strategy of guselkumab (Herranz-Pinto et al., 2023). After an initial complete response, patients re-administered guselkumab only when their absolute PASI reached ≥1. The follow-up was 88 weeks. Patients were divided into four groups: one standard dose group and three groups based on the % DR of the standard dose. The “blue group” had an average reduction of 29% (N = 24), the “orange group” had 52% (N = 10), and the “red group” had 71% (N = 11). All DR groups showed a significant decrease in PASI between weeks 11 and 20 compared to the baseline. After 1 year, drug survival curves showed a survival rate of 93.5% in the overall population (including patients on standard dose), 94.4% in the blue group, and 100% in the orange and red groups without significant differences between groups (*p* = 0.48) (Herranz-Pinto et al., 2023).

#### 3.3.5 van der Schoot et al.,—prospective cohort study (N = 59) on the effectiveness of retreatment with adalimumab, etanercept, and ustekinumab

One prospective cohort study by van der Schoot et al. (2022a) specifically analyzed the effectiveness of retreatment with the standard dose in the case of relapse after DR in 59 patients using either adalimumab (N = 23), etanercept (N = 16), or ustekinumab (N = 20). A total of 40 out of 59 patients (68%) returned to the standard dose based on the protocol (PASI and/or DLQI >5) and 19/59 patients (32%) at their own request. After 1 year of retreatment with the standard dose, the absolute PASI was comparable to the PASI at the start of DR. The median PASI at the start of DR was 2.4 ([interquartile range (IQR) 1.5-3.0]) and the median difference with the PASI after 1 year of retreatment was 0.0 [(IQR −0.8; −1.5)] (van der Schoot et al., 2022a).

### 3.4 Quality of life

Three out of the four included studies testing DR strategies also reported on the QoL. Both studies by Atalay et al. (2021); Atalay et al. (2022b) included the QoL by including the DLQI score, in addition to PASI, in their eligibility criteria (DLQI ≤5), strategy, and as a measurement tool for relapses (DLQI >5) in patients using adalimumab, etanercept, or ustekinumab. In the 1-year extension study, the median (IQR) DLQI scores of the 26 patients who were still on a low dose of adalimumab, etanercept, or ustekinumab at the end of the 1-year CONDOR trial were 1.0 [0.0–3.0] at 12 months, 1.0 [1.0–3.0] at 15 months, 1.0 [0.3–2.0] at 18 months, 0.5 [0.0–1.8] at 21 months, and 1.0 [0.0–1.0] at 24 months (Atalay et al., 2022b). No significant differences in DLQI scores were found between patients on DR vs. the standard dose (Atalay et al., 2022b). In the one-step DR study, analyses on the QoL were performed within a sub-cohort of their original cohort, including patients who started DR ≥ 1 year ago (67/80 patients) (Atalay et al., 2021). At the baseline, 6 months, and 12 months, the median (IQR) DLQI scores were 0 [0–1], 0 [0–1.5], and 0.5 [0–2], respectively (Atalay et al., 2021). Di Altobrando et al. (2022) developed an unvalidated four-question questionnaire on patient-perceived satisfaction. The score could range from 5 to 20, with lower scores indicating less satisfaction. The questionnaire was filled out 3 months after the baseline by patients on DR. Of the patients on 40 mg adalimumab Q3W, 79% (37/47 patients) were completely or very satisfied with their reduced dose, 19% (9/47 patients) were quite satisfied, and 2% (1/47 patients) were unsatisfied. Furthermore, 77% (36/47 patients) felt more healed with their reduced dose (Di Altobrando et al., 2022). Of the patients on 50 mg etanercept Q10D, 63% (10/16 patients) were completely or very satisfied, 31% (5/16 patients) were quite satisfied, 6% (1/16 patients) were unsatisfied with their reduced dose, and 69% (11/16 patients) felt more healed (Di Altobrando et al., 2022). Of the patients on infliximab 5 mg/kg Q10W, 19% (4/21 patients) were completely or very satisfied, 81% (17/21 patients) were quite satisfied, no patients were unsatisfied with their reduced dose, and 76% (16/21 patients) felt more healed (Di Altobrando et al., 2022). Of the patients on 45 mg ustekinumab Q14W, 100% (12/12 patients) were completely or very satisfied with their reduced dose and 67% (8/12 patients) felt more healed (Di Altobrando et al., 2022). The previous review by Michielsens et al. (2021) reported the results of three studies on the QoL. All three studies included the DLQI score to measure the QoL. In the CONDOR trial, the median (IQR) DLQI was 1.0 (0.0–2.0) for patients on DR and 0.0 (0.0–2.0) on standard dose, with a mean difference of 0.8 (95% CI 0.3–1.3) after 1 year (Atalay et al., 2020a). Reich et al. (2020) showed in their RCT on secukinumab (300 mg Q6W vs. standard dose) a significant decrease in the DLQI score of 0.62 (95% CI 0.93–0.31, *p* = 0.0001) after 1 year in patients on DR compared to the standard dose. Fotiadou et al. (2012) showed in their retrospective cohort study on adalimumab a DLQI score of 0 for all patients who used adalimumab Q3W for 30 months (10/14 patients).

### 3.5 Safety

Two out of the 14 included studies focused specifically on the safety of DR in the context of antidrug–antibody (ADA) development in patients on DR. Benzaquen et al. (2022) analyzed retrospectively measured serum drug levels and ADA levels of the past 11 years in the blood of patients on DR of adalimumab (Q3W/Q4W) (N = 7). They showed median serum trough levels of 4.7 μg/mL (range 1.9–12.5) after a median period of 18 months of DR. During the 11 years of DR, no patient had developed relevant ADAs against adalimumab; ADA levels remained <10 μg/ml (Benzaquen et al., 2022). Atalay et al. (2022a) measured serum drug levels and ADA levels in the blood samples from the study population of the CONDOR trial (N = 118), which were collected during the trial. For adalimumab, etanercept, and ustekinumab, serum trough levels significantly decreased as intervals were prolonged. No significant differences in detectable ADA levels between DR and the standard dose of adalimumab were found; as for ustekinumab, ADAs were present in neither the DR nor the standard dose (Atalay et al., 2022a). The four studies on DR strategies also reported safety in terms of adverse or serious adverse events (AEs or SAEs). In the 1-year extension study, 1/26 patients on DR (4%) and 5/62 patients on the standard dose (8%) (N = 44 on the standard dose and N = 18 who returned to the standard dose before the start of the extension phase) reported musculoskeletal complaints (Atalay et al., 2022b). One patient, known to have had a previous episode of arthritis, was newly diagnosed with psoriatic arthritis during DR. One SAE in the DR group (in N = 1) and 12 SAEs in the standard dose group (in N = 5) were reported, but no hospital admissions due to exacerbations took place, and no SAEs were deemed causally related to DR (Atalay et al., 2022b). In the one-step DR study, DR was discontinued due to joint complaints in 2/36 patients (6%); no SAEs related to DR were reported (Atalay et al., 2021). Di Altobrando et al. (2022) mentioned that DR did not result in an increase in AEs. Herranz-Pinto et al. (2023) reported no SAEs related to DR. The previous review by Michielsens et al. (2021) showed the results of six studies on safety. One of these studies reported on the incidence of ADA development in patients on ustekinumab Q24W vs. standard dose and also showed no differences (Blauvelt et al., 2017). Five out of 6 studies showed comparable rates of AEs and/or SAEs between DR and standard dose after a maximal follow-up of 96 weeks (Michielsens et al., 2021). Only in the CONDOR trial was a higher rate of general non-specific musculoskeletal complaints in patients on DR vs. the standard dose reported (rate ratio 4.92; 95% CI 2.04–11.87; *p* < 0.001) (Atalay et al., 2020a). However, none of the studies in the previous review reported safety issues causally related to DR (Michielsens et al., 2021).

### 3.6 Costs

One out of the 14 included studies was specifically about the costs associated with DR. A health-economic evaluation was performed by a cost-utility analysis (CUA) based on CONDOR trial data (Atalay et al., 2020b). The CUA showed a mean difference in the quality-adjusted life years (QALYs; calculated based on specific answers of the Short Form Health Survey (SF-36)) of −0.02 (95th percentile −0.06 to 0.02) and costs of -€3,820 (95th percentile -€3,099 to -€4,509) per patient over 12 months between DR and the standard dose (Atalay et al., 2020b). Two out of the four included studies on DR strategies reported on cost savings. In the one-step DR study by Atalay et al. (2021), cost savings for the 67 patients who started DR ≥1 year ago were analyzed and reported per biologic and for the total DR group. The mean cost savings per patient were €2,919.04 for adalimumab Q3W (N = 37), €1,540.16 for etanercept Q10D (N = 14), €1,579.98 for ustekinumab 45 mg Q18W, and €2,456.29 for 90 mg Q18W (N = 16). After 1 year, absolute cost savings of the total DR group were €159,228.16 compared to the standard dose, representing a mean reduction of 22.7% (Atalay et al., 2021). Di Altobrando et al. (2022) reported cost savings per biologic (€/year/patient on DR): €3,740.65 for adalimumab Q3W (N = 30), €3,489.90 for etanercept Q10D (N = 15), €1,885.80 for infliximab Q10W (N = 16) (based on an average patient of 70 kg with 5 mg/kg), and €1,596.20 for ustekinumab Q14W (N = 9). The previous review by Michielsens et al. (2021) mentioned that cost savings as a result of DR were described in six studies and showed results from five studies. All studies showed cost savings of hundreds to thousands of euros annually when the DR was applied compared to the standard dose (Michielsens et al., 2021).

### 3.7 Uptake and implementation of dose reduction

A total of 6 out of the 14 included studies were specifically focused on the implementation and uptake of DR (Aubert et al., 2022; van der Schoot et al., 2022b; van Muijen et al., 2022; Aubert et al., 2023; van der Schoot et al., 2023a; van der Schoot et al., 2023b). These studies mostly evaluated patients’ or healthcare providers’ experienced barriers or facilitators toward DR through surveys and/or interviews and also included the results of a cohort study, a national consensus study, and an implementation study of a DR protocol. The design and outcomes of these studies are described in detail in Supplemental Appendix S4.

#### 3.7.1 Aubert et al.—report on the uptake of DR in a prospective cohort study (PsoBioTeq registry) (N = 850)

This research study reported on the results of 850 patients in the French prospective PsoBioTeq registry cohort (Aubert et al., 2023). All patients were in remission or had low disease activity (R/LDA) (PASI ≤3 or PGA ≤1 and/or no psoriatic lesions during ≥2 consecutive visits). A total of 93 out of 850 patients started DR by either reducing the dose in mg (N = 6/93; 6%) or interval prolongation (N = 87/93; 94%). The included biologics were TNF-α inhibitors (N = 63/93; 68%), the IL-12/23 inhibitor (N = 22/93; 24%), and IL-17 inhibitors (N = 8/93; 9%). Multivariate analysis showed that the interval from the start of biologic treatment to R/LDA was predictive of starting DR. In particular, patients using TNF-α inhibitors showed that the more rapidly remission was achieved, the sooner DR could be applied, compared to patients using IL-12/23 or IL-17 inhibitors. Age, severity, or type of psoriasis showed no significant impact (Aubert et al., 2023).

#### 3.7.2 van der Schoot et al.—qualitative interviews among psoriasis patients (N = 15)

Qualitative interviews with a total of 15 psoriasis patients using biologics were held about their experience, beliefs, and needs regarding DR (van der Schoot et al., 2023b). The interviews revealed patients’ barriers and facilitators to DR, divided into seven different themes: (1) disease control (the higher the effort needed to reach a low disease activity, the more the patients felt a barrier to start DR); (2) attitudes toward medication and DR (e.g., absence of side effects was a barrier as patients could not see advantages in DR; experiencing side effects was a facilitator, as well as confidence in DR, less medication use, and unpleasant injections); (3) healthcare access and organizational aspects (e.g., quick access to healthcare in the case of relapse was a facilitator of DR); (4) cost reduction (contributing to reduced societal healthcare costs was a facilitator); (5) information needs (adequate information on DR rationale, evidence, expected effectiveness, potential risks, and treatment options in the case of relapse was a facilitator); (6) social aspects (providing patients space to discuss DR with relatives was a facilitator); and (7) decision-making (involving patients in decision-making and the possibility to address patients’ physical and mental health before and during DR were mentioned as a facilitator) (van der Schoot et al., 2023b).

#### 3.7.3 van Muijen et al.—survey on the uptake of DR among dermatologists worldwide (N = 53)

This survey on the uptake of DR was distributed among dermatologists worldwide in 2020 via the International Psoriasis Council and included questions regarding eligibility criteria, strategies, and barriers for applying biologic DR in psoriasis (van Muijen et al., 2022). Fifty-three out of 114 invitees could be included, and 37/53 dermatologists (70%) applied DR. For all IL-17 and IL-23 inhibitors (excluding bimekizumab) DR was applied and most frequently for secukinumab (65%). Also, for the TNF-α inhibitors and IL-12/23 inhibitor DR was applied. The most frequently used criteria for applying DR by the 37/53 “DR-applying dermatologists” were starting DR at the patient’s request (27%), a disease activity score of the absolute PASI or BSA of ≤1 or ≤2 or PGA ≤1 (46%), a minimal treatment duration of ≥1 year (65%), and a stable low disease activity for ≥1 year (41%). DR was most frequently performed in two steps comparable to the strategies shown in Table 1: first, 67% of the standard dose and second, 50%. Additionally, infliximab was not reduced beyond 80% of the standard dose, as shown in Table 1. The discontinuation of DR was most frequently determined by disease activity scores (70%), followed by a combination of disease activity and patients’ requests (24%), solely on patients’ requests (3%) or based on “nothing particular” (3%). In 14/26 dermatologists who used disease activity scores (54%), the dose would be re-increased when the PASI or BSA ≥3; in 13/37 “DR-applying dermatologists” (35%), a clinical evaluation of “moderate disease activity” also resulted in re-increasing the dose, in addition to the use of disease activity scores (van Muijen et al., 2022). Reported barriers for DR by both users and non-users of DR included a lack of scientific evidence on safety and efficacy, lack of guidelines, limited experience with DR and/or prescription of (the newest generation) biologics, time constraints, lack of (technical) support, fear of antibody formation, believing that patients are unwilling to apply DR, and thoughts that biological cost-reducing belongs to pharmaceuticals instead of clinicians. The most frequently reported facilitator to apply DR was cost savings (N = 32/37 “DR-applying dermatologists;” 86%), safety/fewer side effects (43%), patients’ requests (41%), and prevention of unnecessary high dosages (5%) (van Muijen et al., 2022).

#### 3.7.4 Aubert et al.,—survey on the uptake of DR among French dermatologists of the Resopso study group (N = 54)

This survey on the uptake of biologic DR, i.e., investigating strategies used in daily practice, was performed among French dermatologists of the Resopso “Groupe d’Étude Multicentrique” (GEM) study group (Aubert et al., 2022), a community of ≥1,200 French dermatologists and ≥600 other health professionals involved in chronic inflammatory dermatoses (http://resopso.fr) (Resopso). According to the responding dermatologists (N = 54; 5% of the total group), 3 different treatment strategies were adopted in patients with “clear” or “almost clear” psoriasis: stop biologic, DR by interval prolongation, and DR by lowering the administration dose (Aubert et al., 2022). Interval prolongation was proposed as a possible strategy for three out of four IL-17 inhibitors (secukinumab, ixekizumab, and brodalumab), one IL-23 inhibitor (guselkumab), all TNF-α inhibitors, and the IL-12/23 inhibitor. Among the 54 dermatologists, interval prolongation was “most often” (46%) or “always” applied (7%) and stopping biologic use was “often” applied (53%). The most frequently used criteria defining disease activity (clear/almost clear) were DLQI ≤3 (54%), PASI ≤3 (48%), PGA ≤1 (48%), BSA ≤1% (46%), and relative PASI 90 (46%). Different strategies were adopted in the case of relapse after DR: returning to the standard dose (57%), returning to the previous effective dose (15%), applying the induction scheme again (18%), adding another systemic treatment like methotrexate (3%), switch of biologic (2%), or other (5%) (Aubert et al., 2022). Responding dermatologists mentioned the following decision factors that were relevant for applying DR: patient preference (65%), molecule type (54%), low disease activity (50%), immunogenicity risk (50%), age at onset (39%), psoriatic arthritis (39%), biologic non-naivety (35%), risk of loss of efficacy in the case of relapse (35%), risk of relapse (20%), and patient’s age (17%) (Aubert et al., 2022).

#### 3.7.5 van der schoot et al.—national consensus study on DR (N = 27)

An online Delphi procedure (eDelphi) was performed in the Netherlands to achieve consensus among Dutch dermatologists, recruited via the Dutch Association for Dermatology and Venerology, on criteria for biologic DR; 27/850 dermatologists participated (van der Schoot et al., 2022b). Consensus was reached on the following eligibility criteria: a minimal treatment duration of and minimal low disease activity for 6 months; PASI ≤5 and/or PGA 0-2 and DLQI ≤5 at the start of DR; a rheumatologist needs to be consulted prior to DR in the case of psoriatic arthritis; outpatient clinic visits should not become more frequent when DR is applied; and DR (of IL-17 and IL-23 inhibitors) can be considered in individual patients while awaiting more scientific evidence. Consensus was reached on the following DR (dis)continuation criteria: continue DR when PASI ≤5 and/or PGA 0–2 and DLQI ≤5; return to the standard or previous effective dose when PASI >5/PGA >2/DLQI >5 or at patients’ request or when considered necessary by the dermatologists; and consider further DR after 3 months of DR for biologics with a standard interval of <8 weeks and after 6 months for biologics with a standard interval of ≥8 weeks. Regarding the DR strategy, consensus was reached for a two-step DR of first 67% and second 50% of the standard dose, specifically for adalimumab and etanercept, but smaller steps for ustekinumab (van der Schoot et al., 2022b).

#### 3.7.6 van der Schoot et al.—implementation study of a DR protocol in three Dutch hospitals

An implementation study was performed in three Dutch hospitals evaluating the implementation process of a DR protocol for adalimumab, etanercept, and ustekinumab (van der Schoot et al., 2023a). Healthcare providers experienced the following barriers: lack of awareness, knowledge, routine, and experience with DR, time constraints, and lack of (technical) support. Additionally, healthcare providers mentioned the following facilitators: uptake of DR into guidelines, feasible protocols, available additional staff for the support of both physicians and patients to educate and/or support in clinical measurements, involving patients in decision-making, and providing IT solutions regarding automated disease activity scoring systems and decision aids in the electronic health record (van der Schoot et al., 2023a).

## 4 Current research gaps and potential developments

The literature on the newest generation of biologics is scarce, and a substantial number of studies were excluded based on the lack of information needed to compare studies and evaluate DR strategies. For instance, a description of DR strategies and exact dosing schedules was often missing. Improving reporting standards for DR studies would, therefore, be highly valuable. The effectiveness of DR strategies was sometimes not described, but it is essential to evaluate the added value of such interventions. Moreover, study populations are usually small, which makes drawing conclusions more difficult and the results less generalizable. It was interesting that 2 out of the 39 studies performed a DR strategy, which was not explicitly included in the search terms. Sanz-Gil et al. (2020) performed a retrospective cohort study on individualized dosing of the self-administration of biologics in patients with plaque psoriasis. They showed that individualization of dosages according to patients’ needs and their responses resulted in injection interval prolongation but in a way that patients were more in the lead when they thought it was necessary to inject biologics. In most cases, this strategy resulted in an improvement in the PASI score (Sanz-Gil et al., 2020). However, this study was excluded after reading the full text due to a lack of clear effectiveness measurements. Herranz-Pinto et al. (2023) performed a similar DR strategy as patients used guselkumab on-demand, although patients re-administered guselkumab only when the absolute PASI reached ≥1, as previously shown. Gisondi et al. (2022) performed a prospective interventional study on the as-needed administration of risankizumab in 64 patients with plaque psoriasis and showed that patients maintained a PASI < 1 up to 38 weeks after injection. These studies showed that an as-needed DR strategy could also be a promising intervention. Therefore, administration as needed might be a potential development for biologic DR in plaque psoriasis, but a research gap still exists in this topic.

## 5 Discussion

This review provides an overview of the latest literature on biologic DR in plaque psoriasis, of all biologics including the newest generation of biologics and uptake and implementation of DR as new aspects, updating a previous scoping review on biologic DR published in 2021 (Michielsens et al., 2021). Reviewing literature published between 2020 and July 2023 showed that studies on the (cost-)effectiveness and/or safety of biologic DR in psoriasis are still scarce, especially regarding the newest generation biologics IL-17 and IL-23 inhibitors. Only one study on the DR strategy included an IL-23 inhibitor: guselkumab (Herranz-Pinto et al., 2023). Almost all IL-17 and IL-23 inhibitors were included in studies on the uptake and implementation of DR. In total, 14 articles were included (Atalay et al., 2020b; Atalay et al., 2021; Atalay et al., 2022a; Aubert et al., 2022; van der Schoot et al., 2022a; Atalay et al., 2022b; Benzaquen et al., 2022; van der Schoot et al., 2022b; Di Altobrando et al., 2022; van Muijen et al., 2022; Aubert et al., 2023; van der Schoot et al., 2023a; van der Schoot et al., 2023b; Herranz-Pinto et al., 2023). Multiple studies were excluded due to uncertainty in the DR strategy studied, induction scheme, or absence of effect measurements, and specifically, cost studies regarding IL-17 and IL-23 inhibitors did not include DR. Considering the studies on DR strategies, the eligibility criteria for DR mainly included biologic use for ≥6 months, a stable low disease activity from ≥6 months to ≥1 year, determined by an absolute or relative PASI (PASI ≤3/≤5/PASI 75–100) and/or DLQI ≤3/≤5, or BSA ≤1/≤2, or PGA ≤1/0-2 during a period ranging from 12 weeks to ≥1 year (Atalay et al., 2021; Aubert et al., 2022; Atalay et al., 2022b; van der Schoot et al., 2022b; Di Altobrando et al., 2022; van Muijen et al., 2022; Herranz-Pinto et al., 2023). DR was most frequently performed by interval prolongation in two steps: first, 67% of the standard dose, and second, 50% (see also Table 1). The study on DR of guselkumab showed that patients in all DR groups using guselkumab 100 mg Q11W or Q17W or Q27W, had a significant decrease in the PASI between weeks 11 and 20 after the start of DR compared to the baseline (Herranz-Pinto et al., 2023). The other studies on DR strategies showed no significant differences in effectiveness between patients on DR and the standard dose, especially for adalimumab, etanercept, infliximab, and ustekinumab (Atalay et al., 2021; Atalay et al., 2022b; Di Altobrando et al., 2022). In general, in the case of a relapse after DR, retreatment with the standard dose resulted in comparable disease activity as before the start of DR (Atalay et al., 2021; van der Schoot et al., 2022a; Atalay et al., 2022b; Di Altobrando et al., 2022). Regarding AEs and/or SAEs, no differences were found between patients on DR and the standard dose. Some studies even showed less or no AEs/SAEs in DR compared to the standard dose (Atalay et al., 2022b; Di Altobrando et al., 2022), and there were no signs of increased ADA development for adalimumab or ustekinumab (Atalay et al., 2022a; Benzaquen et al., 2022). No safety data on the newest biologics were published. Three studies reported on cost savings; these data were also mainly based on the DR of the first-generation biologics (Atalay et al., 2020b; Atalay et al., 2021; Di Altobrando et al., 2022). Regarding the uptake and implementation of DR, barriers and facilitators were identified that are important to take into account when implementing DR in practice (Aubert et al., 2022; van der Schoot et al., 2022b; van Muijen et al., 2022; Aubert et al., 2023; van der Schoot et al., 2023a; van der Schoot et al., 2023b). This review revealed the variety of DR strategies and showed the large body of evidence on the uptake and implementation of DR. Taking into account the most important facilitators (e.g., adequate information for patients and clear guidelines for dermatologists), as well as finding solutions for substantial barriers (time constraints and lack of support), is crucial. This review also identified potential developments for future research as some recent studies performed dose reduction by administration as needed and also showed promising results. Additionally, as mentioned before, some studies tested a DR strategy in which they reduced the dose from the start of biologics instead of following the induction scheme and also showed that this could be a promising intervention. However, these strategies were outside the scope of this review but might be a topic of added value for future studies. A limitation is that the search included only English-language articles.

The total body of evidence on DR strategies mainly comprised observational studies and RCTs were scarce with underrepresentation of the newest generation biologics. Additionally, a relatively large number of the newly included studies were performed or coordinated by the same study group/center. The diversity of studies could hamper the generalizability of results on the effectiveness, safety, and applicability of DR in different healthcare systems.

In summary, DR studies on TNF-α inhibitors and IL-12/23 inhibitor and several studies on some of the earlier IL-17 inhibitors and the IL-23 inhibitor guselkumab, robustly showed good clinical effectiveness and safety of various DR strategies, as well as the potential for substantial cost-savings. However, the literature on DR strategies of the newest generation of biologics remains scarce, and future research on DR strategies of IL-17 and IL-23 inhibitors remains necessary to complement guidelines on DR as guidelines are critical for DR implementation. Studies on the uptake and implementation of DR of almost all biologics of the first- and newest generation were prevalent, and this review provides an overview of facilitators and barriers for implementing DR. We believe that the implementation of DR in practice can be more successful when taking into account these important factors in implementation strategies.

## References

[B1] Armstrong A. W. Puig L. Joshi A. Skup M. Williams D. Li J. (2020). Comparison of biologics and oral treatments for plaque psoriasis: a meta-analysis. JAMA Dermatol 156, 258–269. 10.1001/jamadermatol.2019.4029 32022825 PMC7042876

[B2] Atalay S. Berends S. Groenewoud H. Mathot R. Njoo D. Mommers J. (2022a). Serum drug levels and anti-drug antibodies in the context of dose tapering by interval prolongation of adalimumab, etanercept and ustekinumab in psoriasis patients: results of the CONDOR trial. J. dermatological Treat. 33, 2680–2684. 10.1080/09546634.2022.2043546 35193441

[B3] Atalay S. Van Den Reek J. Den BROEDER Van Vugt L. J. Otero M. E. Njoo M. D. (2020a). Comparison of tightly controlled dose reduction of biologics with usual care for patients with psoriasis: a randomized clinical trial. JAMA Dermatol 156, 393–400. 10.1001/jamadermatol.2019.4897 32049319 PMC7042801

[B4] Atalay S. Van Den Reek J. Groenewoud J. M. M. Van De Kerkhof P. C. M. Kievit W. De Jong E. (2022b). Two-year follow-up of a dose reduction strategy trial of biologics adalimumab, etanercept, and ustekinumab in psoriasis patients in daily practice. J. Dermatol. Treat. 33, 1591–1597. 10.1080/09546634.2020.1869147 33356686

[B5] Atalay S. Van Den Reek J. Otero M. Njoo M. Mommers J. Ossenkoppele P. (2020b). Health economic consequences of a tightly controlled dose reduction strategy for adalimumab, etanercept and ustekinumab compared with standard psoriasis care: a cost-utility analysis of the CONDOR study. Acta dermato-venereologica 100, adv00340. 10.2340/00015555-3692 33196101 PMC9309701

[B6] Atalay S. Van Der Schoot L. Vandermaesen L. Van Vugt L. Eilander M. Van Den Reek J. (2021). Evaluation of a one-step dose reduction strategy of adalimumab, etanercept and ustekinumab in patients with psoriasis in daily practice. Acta dermato-venereologica 101, adv00463. 10.2340/00015555-3815 33903920 PMC9367038

[B7] Aubert H. Arlegui H. De Rycke Y. Bachelez H. Beylot-Barry M. Dupuy A. (2023). Biologic tapering for patients with psoriasis with low disease activity: data from the French PsoBioTeq Registry. Br. J. dermatology 188, 150–152. 10.1093/bjd/ljac024 36689510

[B8] Aubert H. Mahé E. Fougerousse A. Maccari F. Beneton N. Resopso GEM (2022). Dose spacing and reduction strategies in biotherapies for stable, clear or almost clear psoriasis: a survey of practices in France. Ann. Dermatol Venereol. 149, 68–70. 10.1016/j.annder.2021.07.001 34887084

[B9] Baniandres O. Rodriguez-Soria V. J. Romero-Jimenez R. M. Suarez R. (2015). Dose modification in biologic therapy for moderate to severe psoriasis: a descriptive analysis in a clinical practice setting. Actas Dermosifiliogr. 106, 569–577. 10.1016/j.ad.2015.02.003 25935194

[B10] Bardazzi F. Loi C. Prignano F. Ricceri F. Giordano F. Patrizi A. (2016). Down-titration of infliximab: the real-life use in psoriatic patients. J. Drugs Dermatol 15.

[B11] Benzaquen M. Munshi M. Bossart S. Feldmeyer L. Emelianov V. Yawalkar N. (2022). Long-term dose optimization of adalimumab via dose spacing in patients with psoriasis. Bioeng. Basel 9, 387. Switzerland. 10.3390/bioengineering9080387 PMC9405054 36004912

[B12] Blauvelt A. Ferris L. K. Yamauchi P. S. Qureshi A. Leonardi C. L. Farahi K. (2017). Extension of ustekinumab maintenance dosing interval in moderate-to-severe psoriasis: results of a phase IIIb, randomized, double-blinded, active-controlled, multicentre study (PSTELLAR). Br. J. Dermatol 177, 1552–1561. 10.1111/bjd.15722 28600818

[B13] Di Altobrando A. Magnano M. Offidani A. Parodi A. Patrizi A. Campanati A. (2022). Deferred time of delivery of biologic therapies in patients with stabilized psoriasis leads to a 'perceived satisfaction': a multicentric study. J. Dermatol. Treat. 33, 415–419. 10.1080/09546634.2020.1759769 32314934

[B14] Fotiadou C. Lazaridou E. Sotiriou E. Ioannides D. (2012). Adalimumab for psoriasis in Greece: clinical experience in a tertiary referral centre. J. Eur. Acad. Dermatol Venereol. 26, 1298–1303. 10.1111/j.1468-3083.2011.04290.x 21967627

[B15] Ghoreschi K. Balato A. Enerback C. Sabat R. (2021). Therapeutics targeting the IL-23 and IL-17 pathway in psoriasis. Lancet 397, 754–766. 10.1016/S0140-6736(21)00184-7 33515492

[B16] Gisondi P. Cazzaniga S. Chimenti S. Maccarone M. Picardo M. Girolomoni G. (2015). Latent tuberculosis infection in patients with chronic plaque psoriasis: evidence from the Italian Psocare Registry. Br. J. Dermatol 172, 1613–1620. 10.1111/bjd.13539 25401733

[B17] Gisondi P. Maurelli M. Bellinato F. Girolomoni G. (2022). Is risankizumab as needed administration a good option for patients with plaque psoriasis? J. Eur. Acad. Dermatol Venereol. 36, e713–e715. 10.1111/jdv.18182 35470475

[B18] Hansel K. Bianchi L. Lanza F. Bini V. Stingeni L. (2017). Adalimumab dose tapering in psoriasis: predictive factors for maintenance of complete clearance. Acta Derm. Venereol. 97, 346–350. 10.2340/00015555-2571 27868145

[B19] Herranz-Pinto P. Alonso-Pacheco M. Feltes-Ochoa R. Mayor-Ibarguren A. Servera-Negre G. Busto-Leis J. (2023). Real-world performance of a new strategy for off-label use of guselkumab in moderate to severe psoriasis: super-responder patients as the epitome of efficacy and optimisation. Clin. drug Investig. 43, 517–527. 10.1007/s40261-023-01280-9 PMC10374766 37402097

[B20] Lebwohl M. Strober B. Menter A. Gordon K. Weglowska J. Puig L. (2015). Phase 3 studies comparing brodalumab with ustekinumab in psoriasis. N. Engl. J. Med. 373, 1318–1328. 10.1056/NEJMoa1503824 26422722

[B21] Lee E. B. Thomas L. W. Egeberg A. Wu J. J. (2018). Dosage adjustments in patients with psoriasis on adalimumab - a retrospective chart review. J. Eur. Acad. Dermatol Venereol. 32, e292–e293. 10.1111/jdv.14826 29377299

[B22] Lopez-Ferrer A. Vilarrasa E. Gich I. J. Puig L. (2013). Adalimumab for the treatment of psoriasis in real life: a retrospective cohort of 119 patients at a single Spanish centre. Br. J. Dermatol 169, 1141–1147. 10.1111/bjd.12543 23909993

[B23] Menting S. P. Coussens E. Pouw M. F. Van Den Reek J. M. Temmerman L. Boonen H. (2015). Developing a therapeutic range of adalimumab serum concentrations in management of psoriasis: a step toward personalized treatment. JAMA Dermatol 151, 616–622. 10.1001/jamadermatol.2014.5479 25807311

[B24] Michielsens C. A. J. Van Muijen M. E. Verhoef L. M. Van Den Reek J. De Jong E. (2021). Dose tapering of biologics in patients with psoriasis: a scoping review. Drugs 81, 349–366. 10.1007/s40265-020-01448-z 33453052 PMC7952351

[B25] Piaserico S. Gisondi P. De Simone C. Marinello E. Conti A. Amerio P. (2016). Down-titration of adalimumab and etanercept in psoriatic patients: a multicentre observational study. Acta Derm. Venereol. 96, 251–252. 10.2340/00015555-2209 26270599

[B26] Reich K. Mrowietz U. Radtke M. A. Thaci D. Rustenbach S. J. Spehr C. (2015). Drug safety of systemic treatments for psoriasis: results from the German Psoriasis Registry PsoBest. Arch. Dermatol Res. 307, 875–883. 10.1007/s00403-015-1593-8 26358263 PMC4643107

[B27] Reich K. Puig L. Szepietowski J. Paul C. Lacour J. Tsianakas A. (2020). Secukinumab dosing optimization in patients with moderate-to-severe plaque psoriasis: results from the randomized. open-label OPTIMISE study, Engl. 10.1111/bjd.18143 31102257

[B28] Resopso G. E. M. (2023). Reso dermatology. Available: https://www.reso-dermatologie.fr/ (Accessed December 15, 2023).

[B29] Romero-Jimenez R. M. Escudero-Vilaplana V. Baniandres Rodriguez O. Garcia-Gonzalez X. Sanjurjo Saez M. (2016). Efficiency of biological therapies in patients with moderate to severe psoriasis: impact of a pharmacotherapeutic protocol. J. Dermatol. Treat. 27, 198–202. 10.3109/09546634.2015.1088127 26365424

[B30] Sanz-Gil R. Pellicer A. Montesinos M. C. Valcuende-Cavero F. (2020). Improved effectiveness from individualized dosing of self-administered biologics for the treatment of moderate-to-severe psoriasis: a 5-year retrospective chart review from a Spanish University Hospital. J. Dermatol. Treat. 31, 370–377. 10.1080/09546634.2019.1602246 30924390

[B31] Scherer K. Spoerl D. Bircher A. J. (2010). Adverse drug reactions to biologics. J. Dtsch. Dermatol Ges. 8, 411–426. 10.1111/j.1610-0387.2010.07339.x 20136676

[B32] Snast I. Atzmony L. Braun M. Hodak E. Pavlovsky L. (2017). Risk for hepatitis B and C virus reactivation in patients with psoriasis on biologic therapies: a retrospective cohort study and systematic review of the literature. J. Am. Acad. Dermatol 77, 88–97. 10.1016/j.jaad.2017.01.037 28495497

[B33] Taniguchi T. Noda S. Takahashi N. Yoshimura H. Mizuno K. Adachi M. (2013). An observational, prospective study of monthly adalimumab therapy for disease maintenance in psoriasis patients: a possible new therapeutic option for good responders to the initial induction treatment. J. Eur. Acad. Dermatol Venereol. 27, 1444–1447. 10.1111/j.1468-3083.2012.04610.x 22702802

[B34] Thomaidou E. Ramot Y. (2019). Injection site reactions with the use of biological agents. Dermatol Ther. 32, e12817. 10.1111/dth.12817 30637967

[B35] Van Bezooijen J. S. Van DOORN Schreurs M. W. J. Koch B. C. P. Te Velthuis H. Prens E. P. (2017). Prolongation of biologic dosing intervals in patients with stable psoriasis: a feasibility study. Ther. Drug Monit. 39, 379–386. 10.1097/FTD.0000000000000420 28570371

[B36] Van Der Schoot L. Atalay S. Otero M. Kievit W. Van Den Reek J. De Jong E. (2022a). Regaining adequate treatment responses in patients with psoriasis who discontinued dose reduction of adalimumab. England: etanercept or ustekinumab. 10.1111/bjd.21797 PMC10087531 35895852

[B37] Van Der Schoot L. Baerveldt E. Van Enst W. Menting S. Seyger M. Wanders S. (2022b). National consensus on biologic dose reduction in psoriasis: a modified eDelphi procedure. J. dermatological Treat. 34, 2154570. 10.1080/09546634.2022.2154570 36472386

[B38] Van Der Schoot L. Janssen J. Bastiaens M. De Boer-Brand A. Christiaansen-Smit C. Enomoto D. (2023a). Steps towards implementation of protocolized dose reduction of adalimumab, etanercept and ustekinumab for psoriasis in daily practice. J. dermatological Treat. 34, 2186728. 10.1080/09546634.2023.2186728 PMC10013325 36867069

[B39] Van Der Schoot L. Verhoef L. Van E. E. Van Oort F. Pieterse A. Seyger M. (2023b). Patients' perspectives towards biologic dose reduction in psoriasis: a qualitative study. Archives dermatological Res. 315, 1735–1745. 10.1007/s00403-023-02566-w PMC10338615 36813868

[B40] Van Muijen M. Van Der Schoot L. Van Den Reek J. De Jong E. (2022). Attitudes and behaviour regarding dose reduction of biologics for psoriasis: a survey among dermatologists worldwide. Archives dermatological Res. 314, 687–695. 10.1007/s00403-021-02273-4 PMC9307528 34467442

