# Corrigendum: Dose reduction of biologics in patients with plaque psoriasis: a review

**DOI:** 10.3389/fphar.2024.1505626

**Published:** 2024-10-14

**Authors:** C. A. M. van Riel, C. A. J. Michielsens, M. E. van Muijen, L. S. van der Schoot, J. M. P. A. van den Reek, E. M. G. J. de Jong

**Affiliations:** ^1^ Radboud University Medical Centre, Nijmegen, Netherlands; ^2^ Department of Dermatology, Radboud University Medical Centre, Nijmegen, Gelderland, Netherlands; ^3^ Maastricht University Medical Centre, Maastricht, Limburg, Netherlands; ^4^ Department of Dermatology, Faculty of Health, Medicine and Life Sciences, Maastricht University, Maastricht, Netherlands, Netherlands; ^5^ Radboud University, Nijmegen, Gelderland, Netherlands

**Keywords:** psoriasis, dose reduction, dose tapering, clinical practice, implementation, biologics, biologicals, (cost-)effectiveness

In the published article, there was an error. The symbol ‘≤’, in the phrase “[…] biologic use for ≤6 months […]” is incorrect. This should be the symbol ‘≥’.

A correction has been made to the **Discussion**, Paragraph 1. This sentence previously stated:

“Considering the studies on DR strategies, the eligibility criteria for DR mainly included biologic use for ≤6 months, a stable low disease activity from ≥6 months to ≥1 year, determined by an absolute or relative PASI (PASI ≤3/≤5/PASI 75–100) and/or DLQI ≤3/≤5, or BSA ≤1/≤2, or PGA ≤1/0-2 during a period ranging from 12 weeks to ≥1 year (Atalay et al., 2021; Aubert et al., 2022; Atalay et al., 2022b; van der Schoot et al., 2022b; Di Altobrando et al., 2022; van Muijen et al., 2022; Herranz-Pinto et al., 2023).”

The corrected sentence appears below:

“Considering the studies on DR strategies, the eligibility criteria for DR mainly included biologic use for ≥6 months, a stable low disease activity from ≥6 months to ≥1 year, determined by an absolute or relative PASI (PASI ≤3/≤5/PASI 75–100) and/or DLQI ≤3/≤5, or BSA ≤1/≤2, or PGA ≤1/0-2 during a period ranging from 12 weeks to ≥1 year (Atalay et al., 2021; Aubert et al., 2022; Atalay et al., 2022b; van der Schoot et al., 2022b; Di Altobrando et al., 2022; van Muijen et al., 2022; Herranz-Pinto et al., 2023).”

The authors apologize for this error and state that this does not change the scientific conclusions of the article in any way. The original article has been updated.

