# Supplementary material for: Dose reduction of biologics in patients with plaque psoriasis: a review
Source: Front Pharmacol. 2024 Mar 28;15:1369805. doi: 10.3389/fphar.2024.1369805 (PMC11007084; doi:10.3389/fphar.2024.1369805)
Supplement: Supplementary file 1 [file DataSheet2.PDF]

# Supplemental appendix 4: Detailed overview of studies on uptake and implementation of dose reduction

A total of six out of the 14 included studies were specifically focused on implementation and uptake of DR.(van Muijen et al., 2022, Aubert et al., 2022, Aubert et al., 2023, van der Schoot et al., 2022, van der Schoot et al., 2023a, van der Schoot et al., 2023b) These studies mostly evaluated patients' or healthcare providers' experienced barriers or facilitators towards DR by surveys and/or interviews and also included results of a cohort study, a national consensus study and an implementation study of a DR protocol.

### *Aubert et al., – report on uptake of DR in prospective cohort study (PsoBioTeq Registry) (N=850)*

This research letter reported on the results of 850 out of the 2427 included patients in the French prospective PsoBioTeq Registry cohort.(Aubert et al., 2023) All patients were in remission or had low disease activity (R/LDA) (PASI ≤3 or PGA ≤1 and/or no psoriatic lesions during ≥2 consecutive visits). A total of 93 out of 850 patients started DR by either reducing dose in mg (N=6/93; 6%) or interval prolongation (N=87/93; 94%). Included biologics were TNF-α inhibitors (N=63/93; 68%), the IL-12/23 inhibitor (N=22/93; 24%), and IL-17 inhibitors (N=8/93; 9%). DR was applied after a median of 25.5 months after start of biologic. Multivariate analysis showed that the interval from start of biologic treatment to R/LDA was predictive of starting DR (subdistribution hazard ratio 0.96 (95% CI 0.93-0.99), p=0.016). Especially patients using TNF-α inhibitors showed that the more rapidly remission was achieved, the sooner DR could be applied, compared to patients using IL-12/23 or IL-17 inhibitors. Patients' age, severity or type of psoriasis showed no significant impact.(Aubert et al., 2023)

### *Van der Schoot et al., – qualitative interviews among psoriasis patients (N=15)*

Qualitative interviews with a total of 15 psoriasis patients using biologics were held, with both positive and negative experiences with DR, questioning about their experience, beliefs and needs regarding DR.(van der Schoot et al., 2023b) The interviews revealed patients' barriers and facilitators to DR divided in seven different themes which will be described per theme. First, disease control. The higher the effort needed to reach a low disease activity, the more patients felt a barrier to start DR. Therefore DR could best be started after a substantial period of biologic use with adequate disease control, gaining trust in the effect of DR and also reducing fear of relapse. Attitudes towards medication and DR was a second identified theme: Not experiencing side effects was seen as a barrier as patients could not see advantages in DR, while experiencing side effects was seen as a facilitator. Patients mentioned that confidence in DR effects and in regaining adequate disease control after returning to the previous effective/standard dose was very important prior to start. Less medication use, less (unpleasant) injections, and less pharmacy visits/delivery moments were also mentioned as facilitators of DR. Third, healthcare access and organizational aspects. According to patients, the higher the number of different healthcare providers involved in a patient's treatment, the more limited the application of DR could be as the estimation of a patient's eligibility for DR could be hampered. Quick access to healthcare in case of relapse and monitoring of disease activity were important facilitators of DR. Fourth, cost reduction. Although patients have no individual financial advantages of DR due to the fixed amount for healthcare insurance in the Netherlands, patients mentioned contributing to reduced societal healthcare costs due to DR as a facilitator, hopefully increasing biologic availability. Fifth, information needs. Information on DR rationale, evidence, expected effectiveness, potential risks and treatment

options in case of relapse should be presented very early during an outpatient visit and preferably with supplemental written information, according to patients. Sixth, social aspects. Patients mentioned that the possibility to discuss the option of DR with relatives is important and that they are allowed to think and decide by themselves. Therefore, healthcare providers also need to take time and listen to patients. Seventh, decision-making. Every step in the process of DR should be discussed with the patient. This involves the patient in decision-making and the possibility to address patients' physical and mental health, thereby gaining trust and personalizing treatment.(van der Schoot et al., 2023b)

*Van Muijen et al., – survey on uptake of DR among dermatologists worldwide (N=53)*
This survey on uptake of DR was distributed among dermatologists worldwide in 2020 via the International Psoriasis Council and included questions regarding eligibility criteria, strategies and barriers for applying biologic DR in psoriasis.(van Muijen et al., 2022) A total of 57 dermatologists out of 114 invitees responded of which 4/57 dermatologists did not prescribe biologics and were excluded from analysis. From the 53 included dermatologists, 37/53 dermatologists (70%) applied DR. Patients agreed "often" or "always" with start of DR, according to 29/37 'DR applying dermatologists' (78%). DR was most frequently applied in patients using adalimumab (N=28/37; 76%), etanercept (N=19/37; 51%), ustekinumab (N=19/37; 51%), and secukinumab (N=24/37; 65%) but also in infliximab, certolizumab, ixekizumab, brodalumab, guselkumab, risankizumab, and tildrakizumab. Disease activity was mainly measured by PASI (N=46/53; 87%), Body Surface Area (BSA) (N=42/53; 79%) and PGA (N=28/53; 53%). Only 6/53 dermatologists used other tools like DLQI or VAS scores. The most frequently used criteria for applying DR by the 37/53 'DR applying dermatologists' were: starting DR at patient's request (N=10/37; 27%), a disease activity score of absolute PASI or BSA of ≤1 or ≤2 or PGA ≤1 (N=17/37; 46%), a minimal treatment duration of ≥1 year (N=24/37; 65%) and a stable low disease activity for ≥1 year (N=15/37; 41%). Other frequently mentioned criteria were a minimal treatment duration of ≥6 months and a stable low disease activity for ≥3 or ≥6 months. DR was most frequently performed in two steps comparable to the strategies shown in Table 1: firstly 67% of standard dose, secondly 50%. Additionally, infliximab was not reduced beyond 80% of the standard dose, as shown in Table 1. Discontinuation of DR was most frequently determined by disease activity scores (N=26/37; 70%) followed by a combination of disease activity and patients' request (N=9/37; 24%), solely on patients' request (N=1/37; 3%) or based on 'nothing particular' (N=1/37; 3%). In 14/26 dermatologists who used disease activity scores (54%), the dose would be re-increased when PASI or BSA ≥3. Other criteria mentioned for re-increasing the dose were BSA >0% or PGA >0 (N=1/26; 4%), or BSA >10% (N=1/26; 4%). In 13/37 'DR applying dermatologists' (35%), a clinical evaluation of 'moderate disease activity' also resulted in re-increasing the dose, additional to the use of disease activity scores.(van Muijen et al., 2022)

Barriers against application of DR were mostly similar among dermatologists that did not apply DR (N=16/53; 30%) and those who did apply DR but would like to apply it more often (N=21/37; 57%). These barriers included lack of scientific evidence on safety and efficacy, lack of guidelines, limited experience with DR and/or prescription of (the newest generation) biologics, time constraints, lack of (technical) support, fear for antibody formation, believing that patients are unwilling to DR and thoughts that biological cost-reducing belongs to pharmaceuticals instead of clinicians. The biggest difference between the groups was the potential risk of psoriasis relapse, as this was the most frequent reason in 9/16 dermatologists who did not apply DR (56%) and only mentioned by 1/21 'DR applying dermatologists' (5%). The most frequent reported facilitator to apply DR was cost savings (N=32/37 'DR applying dermatologists'; 86%). Other reported facilitators were safety/less side effects (N=16/37; 43%), patients' request (N=15/37; 41%) and preventing the use of unnecessary high dosages (N=2/37; 5%).(van Muijen et al., 2022)

*Aubert et al., – survey on uptake of DR among French dermatologists of the Resopso study group (N=54)*

This survey on uptake of biologic DR, i.e. investigating strategies used in daily practice, was performed among French dermatologists of the Resopso 'Groupe d'Étude Multicentrique' (GEM) study group (Aubert et al., 2022), a community of ≥1200 French dermatologists and ≥600 other health professionals involved in chronic inflammatory dermatoses (http://resopso.fr).(Resopso) According to the responding dermatologists (N=54; 5% of total group), three different treatment strategies were adopted in patients with 'clear' or 'almost clear' psoriasis: stop biologic, DR by interval prolongation, and DR by lowering the administration dose.(Aubert et al., 2022) Interval prolongation was proposed as a possible strategy for three out of four IL-17 inhibitors (secukinumab, ixekizumab, brodalumab), one IL-23 inhibitor (guselkumab) and all TNFα-inhibitors and IL-12/23 inhibitor. Among the 54 dermatologists, interval prolongation was 'most often' (N=25; 46%) or 'always' applied (N=4; 7%) and stopping biologic use was 'often' applied (N=29; 53%). The most frequently used criteria defining disease activity (clear/almost clear) were DLQI ≤3 (N=29/54; 54%), PASI ≤3 (N=26/54; 48%), PGA ≤1 (N=26/54; 48%), BSA ≤1% (N=25/54; 46%) and relative PASI 90 (N=25/54; 46%). Different strategies were adopted in case of relapse after DR: returning to standard dose (N=31/54; 57%), returning to previous effective dose (N=8/54; 15%), applying the induction scheme again (N=9/54; 18%), adding another systemic treatment like methotrexate (N=2/54; 3%), switch of biologic (N=1/54; 2%), or other (N=3/54; 5%).(Aubert et al., 2022)

Regarding barriers and facilitators of applying DR, the responding dermatologists took the following decision factors into account which could be possible barriers and/or facilitators to apply DR: patient preference (N=35/54; 65%), molecule type (N=29/54; 54%), low disease activity (N=27/54; 50%), immunogenicity risk (N=27/54; 50%), age at onset (N=21/54; 39%), psoriatic arthritis (N=21/54; 39%), biologic non-naivety (N=19/54; 35%), risk of loss of efficacy in case of relapse (N=19/54; 35%), risk of relapse (N=11/54; 20%), and patient's age (N=9/54; 17%).(Aubert et al., 2022)

*Van der Schoot et al., – national consensus study on DR (N=27)*

A national online Delphi procedure (eDelphi) was performed in the Netherlands aiming to achieve consensus among Dutch dermatologists on criteria for biologic DR.(van der Schoot et al., 2022) Dermatologists were recruited by the Dutch Association for Dermatology and Venerology; statements regarding criteria for eligibility, (dis)continuation and strategy of DR were rated. In total, 27/850 dermatologists participated. Consensus regarding the 15 statements was reached when ≥70% of all voters agreed and <15% disagreed. In summary, consensus was reached on the following eligibility criteria: a minimal treatment duration of and minimal low disease activity for 6 months, a PASI ≤5 and/or PGA 0-2 and DLQI ≤5 at start of DR, a rheumatologist needs to be consulted prior to DR in case of psoriatic arthritis, outpatient clinic visits should not become more frequent when DR is applied, DR (of IL-17 and IL-23 inhibitors) can be considered in individual patients while awaiting more scientific evidence. Consensus was also reached on the following DR (dis)continuation criteria: continue DR when PASI ≤5 and/or PGA 0-2 and DLQI ≤5, return to standard or previous effective dose when PASI >5/PGA >2/DLQI >5 or at patients' request or when considered necessary by the dermatologists, consider further DR after 3 months of DR for biologics with standard interval <8 weeks and after 6 months for biologics with standard interval ≥8 weeks. Regarding DR strategy, consensus was reached for two-step DR of firstly 67% and secondly 50% of standard dose specifically for adalimumab and etanercept. Dermatologists suggested after the first voting round intermediate steps regarding DR of ustekinumab leading to consensus on a four-step DR of firstly 80%, secondly 67%, thirdly 57% and fourthly 50%, both for 45mg and 90mg, which is comparable to the shown DR strategy in Table 1. Based on

the abovementioned consensus, an algorithm for DR including DR strategies for adalimumab, etanercept and ustekinumab was developed.(van der Schoot et al., 2022)

*Van der Schoot et al., – implementation study of a DR protocol in three Dutch hospitals*
An implementation study was performed in three Dutch hospitals evaluating the implementation process of a DR protocol for adalimumab, etanercept and ustekinumab, and exploring new factors for optimization of implementation.(van der Schoot et al., 2023a) Participating healthcare providers of the participating hospitals were involved in feedback meetings and qualitative interviews focusing on the implementation strategy, acceptability, feasibility and complexity of the DR protocol. Healthcare providers experienced the following barriers: lack of awareness, knowledge, routine and experience with DR, time constraints, and lack of (technical) support. Additionally, healthcare providers mentioned the following factors that could facilitate implementation of DR: uptake of DR into guidelines, feasible protocols, available additional staff for support of both physicians and patients to educate and/or support in clinical measurements, involving patients in decision-making and providing IT solutions regarding automated disease activity scoring systems and decision aids in the electronic health record.(van der Schoot et al., 2023a)

**References in supplemental 4**


AUBERT, H., ARLEGUI, H., DE RYCKE, Y., BACHELEZ, H., BEYLOT-BARRY, M., DUPUY, A., JOLY, P., JULLIEN, D., MAHÉ, E., PAUL, C., RICHARD, M., SBIDIAN, E., VIGUIER, M., CHOSIDOW, O., TUBACH, F. & BÉNÉTON, N. 2023. Biologic tapering for patients with psoriasis with low disease activity: data from the French PsoBioTeq Registry. *The British journal of dermatology,* 188**,** 150-152.

AUBERT, H., MAHÉ, E., FOUGEROUSSE, A., MACCARI, F. & BENETON, N. 2022. *Dose spacing and reduction strategies in biotherapies for stable, clear or almost clear psoriasis: A survey of practices in France,* France.

RESOPSO, G. E. M. *Reso dermatology* [Online]. Reso dermatology. Available: https://www.reso-dermatologie.fr/ [Accessed 15 December 2023].

VAN DER SCHOOT, L., BAERVELDT, E., VAN ENST, W., MENTING, S., SEYGER, M., WANDERS, S., VAN EE, I., PIETERSE, A., VAN DEN REEK, J. & DE JONG, E. 2022. National consensus on biologic dose reduction in psoriasis: a modified eDelphi procedure. *The Journal of dermatological treatment***,** 2154570.

VAN DER SCHOOT, L., JANSSEN, J., BASTIAENS, M., DE BOER-BRAND, A., CHRISTIAANSEN-SMIT, C., ENOMOTO, D., HOVINGH, R., TUPKER, R., SEYGER, M., VERHOEF, L., VAN DEN REEK, J. & DE JONG, E. 2023a. Steps towards implementation of protocolized dose reduction of adalimumab, etanercept and ustekinumab for psoriasis in daily practice. *The Journal of dermatological treatment,* 34**,** 2186728.

VAN DER SCHOOT, L., VERHOEF, L., VAN EE, I., VAN OORT, F., PIETERSE, A., SEYGER, M., DE JONG, E. & VAN DEN REEK, J. 2023b. Patients' perspectives towards biologic dose reduction in psoriasis: a qualitative study. *Archives of dermatological research*.

VAN MUIJEN, M., VAN DER SCHOOT, L., VAN DEN REEK, J. & DE JONG, E. 2022. Attitudes and behaviour regarding dose reduction of biologics for psoriasis: a survey among dermatologists worldwide. *Archives of dermatological research,* 314**,** 687-695.
