# Supplementary material for: Dose reduction of biologics in patients with plaque psoriasis: a review
Source: Front Pharmacol. 2024 Mar 28;15:1369805. doi: 10.3389/fphar.2024.1369805 (PMC11007084; doi:10.3389/fphar.2024.1369805)
Supplement: Supplementary file 2 [file Table1.PDF]

Supplementary Appendix 1: Full electronic search in PubMed

**Supplementary Table 1. Full electronic search strategy as performed in PubMed on 4 July 2023**
(psoriasis [Majr] OR<br>psoriasis [ti] OR<br>psoriases [ti])
AND
(biological therapy [Mesh:NoExp] OR<br>biological products [Mesh:NoExp] OR<br>biosimilar pharmaceuticals [Mesh] OR<br>Tumor Necrosis Factor Inhibitors [Mesh] OR<br>adalimumab [MeSH] OR<br>certolizumab pegol [MeSH] OR<br>etanercept [MeSH] OR<br>infliximab [MeSH] OR<br>SB2 infliximab [Supplementary Concept] OR<br>ustekinumab [Mesh] OR<br>secukinumab [Supplementary Concept] OR<br>ixekizumab [Supplementary Concept] OR<br>brodalumab [Supplementary Concept] OR<br>bimekizumab [Supplementary Concept] OR<br>risankizumab [Supplementary Concept] OR<br>guselkumab [Supplementary Concept] OR<br>tildrakizumab [Supplementary Concept] OR<br>Interleukin 12/antagonists and inhibitors [Mesh] OR<br>Interleukin 12 subunit p40 [Mesh] OR<br>Interleukin-23/antagonists and inhibitors [Mesh] OR<br>Interleukin 23 subunit p19 [Mesh] OR<br>Interleukin-17/antagonists and inhibitors [Mesh] OR<br>biologic* [tiab] OR<br>biosimilar* [tiab] OR<br>“TNF Antagonist*” [tiab] OR<br>“TNF Inhibitor*” [tiab] OR<br>“TNF Blocker*” [tiab] OR<br>“Tumor Necrosis Factor Antagonist*” [tiab] OR<br>“Tumor Necrosis Factor-a Antagonist*” [tiab] OR<br>“Tumor Necrosis Factor-alpha Antagonist*” [tiab] OR<br>“Tumor Necrosis Factor-alfa Antagonist*” [tiab] OR<br>“Tumor Necrosis Factor Blocker*” [tiab] OR<br>“Tumor Necrosis Factor-a Blocker*” [tiab] OR<br>“Tumor Necrosis Factor-alpha Blocker*”[tiab] OR<br>“Tumor Necrosis Factor-alfa Blocker*”[tiab] OR<br>“Tumor Necrosis Factor blocking agent*” [tiab] OR<br>“Tumor Necrosis Factor Inhibitor*” [tiab] OR<br>“Tumor Necrosis Factor-a Inhibitor*” [tiab] OR<br>“Tumor necrosis factor-alpha inhibitor*” [tiab] OR

"Tumor necrosis factor-alfa inhibitor*" [tiab] OR
"anti tumor necrosis factor agent*" [tiab] OR
"Tumour Necrosis Factor Antagonist*" [tiab] OR
"Tumour Necrosis Factor-a Antagonist*" [tiab] OR
"Tumour Necrosis Factor-alpha Antagonist*" [tiab] OR
"Tumour Necrosis Factor-alfa Antagonist*" [tiab] OR
"Tumour Necrosis Factor Blocker*" [tiab] OR
"Tumour Necrosis Factor-a Blocker*" [tiab] OR
"Tumour Necrosis Factor-alpha Blocker*"[tiab] OR
"Tumour Necrosis Factor-alfa Blocker*"[tiab] OR
"Tumour Necrosis Factor blocking agent*" [tiab] OR
"Tumour Necrosis Factor Inhibitor*" [tiab] OR
"Tumour Necrosis Factor-a Inhibitor*" [tiab] OR
"Tumour necrosis factor-alpha inhibitor*" [tiab] OR
"Tumour necrosis factor-alfa inhibitor*" [tiab] OR
"Anti Tumour necrosis factor agent*" [tiab] OR
"TNF Antagonist*" [tiab] OR
"TNF Inhibitor*" [tiab] OR
"TNF Blocker*" [tiab] OR
"TNF-a Antagonist*" [tiab] OR
"TNF-a Inhibitor*" [tiab] OR
"TNF-a Blocker*" [tiab] OR
"TNF-alpha Antagonist*" [tiab] OR
"TNF-alfa Antagonist*" [tiab] OR
"TNF-alpha Inhibitor*" [tiab] OR
"TNF-alfa Inhibitor*" [tiab] OR
"TNF-alpha Blocker*" [tiab] OR
"TNF-alfa Blocker*" [tiab] OR
adalimumab [tiab] OR
amgevita [tiab] OR
amjevita [tiab] OR
cyltezo [tiab] OR
halimatoz [tiab] OR
hulio [tiab] OR
humira [tiab] OR
hefiya [tiab] OR
hyrimoz [tiab] OR
Idacio [tiab] OR
Imraldi [tiab] OR
solymbic [tiab] OR
trudexa [tiab] OR
certolizumab* [tiab] OR
cimzia [tiab] OR
etanercept [tiab] OR
enbrel [tiab] OR
erelzi [tiab] OR
benepali [tiab] OR
tunex [tiab] OR
infliximab [tiab] OR
flixabi [tiab] OR

inflectra [tiab] OR
remicade [tiab] OR
remsima [tiab] OR
zessly [tiab] OR
ixifi [tiab] OR
avakine [tiab] OR
renflexis [tiab] OR
“Interleukin 12/23 p40” [tiab] OR
“IL 12 inhibitor*” [tiab] OR
“IL 23 inhibitor*” [tiab] OR
“IL 17 inhibitor*” [tiab] OR
“Interleukin 12 inhibitor*” [tiab] OR
“Interleukin 23 inhibitor*” [tiab] OR
“Interleukin 17 inhibitor*” [tiab] OR
“IL 12 blocker*” [tiab] OR
“IL 23 blocker*” [tiab] OR
“IL 17 blocker*” [tiab] OR
“Interleukin 12 blocker*” [tiab] OR
“Interleukin 23 blocker*” [tiab] OR
“Interleukin 17 blocker*” [tiab] OR
“IL 12 antagonist*” [tiab] OR
“IL 23 antagonist*” [tiab] OR
“IL 17 antagonist*” [tiab] OR
“Interleukin 12 antagonist*” [tiab] OR
“Interleukin 23 antagonist*” [tiab] OR
“Interleukin 17 antagonist*”[tiab] OR
“Anti IL 12” [tiab] OR
“Anti IL 23” [tiab] OR
“Anti IL 17*” [tiab] OR
“Anti IL12/23” [tiab] OR
“IL12/23 inhibitor*” [tiab] OR
“IL12/23 blocker*” [tiab] OR
“IL12/23 antagonist*” [tiab] OR
“Interleukin 12 subunit p40” [tiab] OR
“Interleukin 23 subunit p19” [tiab] OR
ustekinumab [tiab] OR
stelara [tiab] OR
secukinumab [tiab] OR
cosentyx [tiab] OR
ixekizumab [tiab] OR
taltz [tiab] OR
brodalumab [tiab] OR
kyntheum [tiab] OR
siliq [tiab] OR
bimekizumab [tiab] OR
bimzelx [tiab] OR
risankizumab [tiab] OR
skyrizi [tiab] OR
guselkumab [tiab] OR
tremfya [tiab] OR

tildrakizumab [tiab] OR<br>ilumetri [tiab] OR<br>ilumya [tiab])
AND
(dose-response relationship, drug [MeSH:NoExp] OR<br>withholding treatment [Mesh:NoExp] OR<br>retreatment [Mesh:NoExp] OR<br>down titrat* [tiab] OR<br>intermittent [tiab] OR<br>interval* [tiab] OR<br>low dos* [tiab] OR<br>lower dos* [tiab] OR<br>reduc* [tiab] OR<br>restart* [tiab] OR<br>retreat* [tiab] OR<br>re-treat* [tiab] OR<br>spacing [tiab] OR<br>sparing [tiab] OR<br>spare* [tiab] OR<br>taper* [tiab])
AND
(2020/01:3000/01[DP] OR 2020/01:3000/01[MHDA] OR 2020/01:3000/01[CRDT])
