# Supplementary material for: Dose reduction of biologics in patients with plaque psoriasis: a review
Source: Front Pharmacol. 2024 Mar 28;15:1369805. doi: 10.3389/fphar.2024.1369805 (PMC11007084; doi:10.3389/fphar.2024.1369805)
Supplement: Supplementary file 3 [file Table2.PDF]

Supplementary appendix 2 (part A and B)

Supplementary Table 2, part A. Data extraction table of the included studies on biologic dose reduction (DR) in adult patients with plaque psoriasis

**Author, year**	Study design	Follow-up[a]	Biologic	N	Treatment duration prior to DR	Eligibility criteria for DR	DR strategy	Retreatment strategy	Time to relapse	Efficacy after retreatment	Outcomes on treatment success
**Articles**											
**Atalay et al., 2022 [14]**	Prospective cohort, observational (UC) (a 12-month open-label extension of the CONDOR study (see Atalay et al., 2020 at top of part B))	12 months	ADA, ETN, UST	88 (44 DR, 44 standard dosing, as initially random-ized in CONDOR study)	≥6 months	PASI and DLQI ≤5 at baseline, with PASI ≤5 during ≥6 months prior to baseline	Stepwise prolongation to: ADA Q3W-Q4W ETN Q10D-Q2W UST Q18W-Q24W – second prolongation (first step at baseline) after 3 months if PASI and DLQI ≤5	Retreatment with previous effective dose if: PASI score >5 and/or DLQI score >5	-	After 24 months a total of 4 out of 16 patients (DR N=2 out of N=10, standard dosing N=2 out of N=6) did not regain PASI and/or DLQI <5.	18 out of the 26 patients who were on DR at end of the CONDOR study (69%) maintained DR at end of the extension phase (N=1 maintained DR at own request while PASI/DLQI >5). N=11 were taking 50% of the original dose and N=7 were taking 67% of the original dose at end of extension phase. Median [IQR] PASI & DLQI scores of patients on DR during extension phase: 12 months: 3.6 [2.6-4.4] & 1.0 [0.0-3.0], 15 months: 3.5 [1.6-4.8] & 1.0 [1.0-3.0], 18 months: 3.0 [1.9-4.0] & 1.0 [0.3-2.0], 21 months: 2.3 [1.5-3.4] & 0.5 [0.0-1.8], 24 months: 1.8 [0.7-3.1] & 1.0 [0.0-1.0]

**Atalay et al., 2021 [15]**	Prospective cohort, observational (UC)	12 months	ADA, ETN, UST	80 (all DR)	≥6 months	PASI and DLQI ≤5 at baseline, with PASI ≤5 during ≥6 months prior to baseline. Participation on patients' request despite PASI or DLQI >5 or treatment <6 months was allowed after approval from their treating physician.	Fixed interval:<br>ADA Q3W<br>ETN Q10D<br>UST Q18W	Retreatment with previous effective dose or standard dose if: PASI and/or DLQI >5. Patients were also able to return to previous effective dose at their own request.	Overall median time to relapse was 19 months (95% CI 14.9-23.1). Split per biologic:<br>ADA 9 months (95% CI 14.7-23.3),<br>UST 19 months (95% CI 12.0-26.0),<br>For ETN this could not be calculated (>50% was still active in survival curve at end of analysis).	In total 8 out of 80 patients (10%) relapsed during follow-up.<br>PASI <5 was regained within 6 months in:<br>- 75% (N=3 out of N=4) of patients who relapsed and returned to standard dose<br>- 100% (N= 4 out of N=4) of patients who relapsed and continued DR at own request	45% (N=36) of total population (N=80) discontinued DR.<br>Split per biologic:<br>ADA 45% (N=19 out of N=42)<br>ETN 44% (N=7 out of N=16)<br>UST 46% (N=10 out of N=22).<br>Reasons for discontinuing DR: patients' experienced reduced effectiveness (N=18; 50%), both patients' and physicians' experienced reduced effectiveness (N=9; 25%), joint complaints (N=2; 6%), another reason/missing (N=7; 19%).
**Di Altebrando et al., 2022 [16]**	Prospective cohort, multicentric, observational (UC)	±102 months (maximal)	ADA, ETN, IFX, UST	199 (96 DR, 103 standard dosing)	≥12 months	PASI 75-100 for ≥12 months	Fixed interval:<br>ADA Q3W<br>ETN Q10D<br>IFX Q10W<br>UST Q14W	Retreatment with standard dose if: worsening of PASI ≥50% of initial value. Patients on standard dose who relapsed, received additional methotrexate 15mg/week (ADA), or 10mg/week or	Relapses occurred after (on average) (DR vs. standard dosing):<br>ADA 10 months vs. 12 months<br>ETN 27 months vs. 65 months<br>IFX 31 months vs. 77 months<br>UST 17 months vs. 40 months.	96% (N=25 out of N=26) of patients on DR who relapsed, were treated with the standard dose and regained effectiveness of the drug. One relapsed patient (on DR of UST) received additional methotrexate	% patients who relapsed (≥50% worsening of initial PASI) (DR vs. standard dosing):<br>ADA N=17/47 (36%) vs. N=9/34 (26%)<br>ETN N=1/16 (6%) vs. N=8/25 (32%)<br>IFX N=5/21 (24%) vs. N=4/7 (57%)<br>UST N=3/12 (25%) vs. N=6/37 (16%)

								ciclosporin 5mg/kg/day (ETN). N=1 on DR who relapsed (UST), received additional methotrexate 10mg/week.	The difference in number of relapsed patients between DR and standard dose was insignificant (p=0.445)	10mg/week and showed significant clinical improvement. All patients on standard dose of ADA (N=9) and ETN (N=8) who relapsed and treated with adjuvant treatment showed significant clinical improvement	
**Herranz-Pinto et al., 2023 [17]**	Retrospective, observational (UC)	90 weeks	GUS	69 (45 DR, 24 standard dosing	≥150 days (± ≥5 months)	PASI 100 after 3 GUS injections (=after 12 weeks)	Dose adjustments were on-demand and classified into groups of reduced doses compared to the standard dose and labelled them:<br>Blue group (>20% <40%; N=24),<br>Orange group (>40% <60%; N=10),<br>Red group (>60%; N=11)	As the dose regimen was on-demand in which patients administered guselkumab if PASI ≥1, retreatment was not specifically analysable.	Drug survival rates (at week 52):<br>Overall population: 93.5%,<br>Standard dose: 86.7%, Blue group: 94.4%, Orange and red groups: 100%.<br>There were no significant differences in survival rates at week 52 (p=0.4872) (event = discontinuation of DR due to an adverse event, lack of efficacy or patient death)	-	The blue group had an average dose reduction of 29% (injections Q11W). Mean (±SD) baseline PASI: 11.9±7.2 PASI between: weeks 11-20: 4.2±3.2 (p<0.0001), weeks 31-50: 3.3±4.2 (p=0.97), weeks 71-90: 2.1±3.0 (p=0.91).<br>The orange group had an average reduction of 52% (Q17W). Mean (±SD) baseline PASI: 10.2±6 PASI between: weeks 11-20: 3.4±3.6 (p=0.002), weeks 31-50: 2.4±3.0 (p=0.98), weeks 71-90: 3.6±0.5 (p=0.99).<br>The red group had an average reduction of 71% (Q27W). Mean (±SD) baseline PASI: 7.2±5.4 PASI between: weeks 11-20: 0.6±1.3 (p=0.003), weeks 31-50: 0.6±0.9 (p>0.99), weeks 71-90: 2.7±3.9 (p=0.95)

Supplementary Table 2, part B. Data extraction table of the previously included studies in the scoping review on biologic dose reduction (DR) in adult patients with plaque psoriasis by Michielsens et al., [13]

**Author, year**	Study design	Follow-up[a]	Biologic	N	Treatment duration prior to DR	Eligibility criteria for DR	DR strategy	Retreatment strategy	Time to relapse	Efficacy after retreatment	Outcomes on treatment success
**Articles**											
**Atalay et al., 2020 [9]**	Randomized controlled trial, open-label CONDOR	12 months	ADA, ETN, UST	111 (53 DR, 58 standard dosing)[b]	≥6 months	PASI and DLQI ≤5 at baseline, with PASI ≤5 during ≥6 months prior to baseline	Stepwise prolongation to:<br>ADA Q3W-Q4W<br>ETN Q10D-Q2W<br>UST Q18W-Q24W<br>Second prolongation (first step at baseline) after 3 months if PASI and DLQI ≤5	Retreatment with previous effective dose if: PASI score >5 and/or DLQI score >5	The first persistent flare occurred after 7.5 months in the DR group vs. after 3 months in standard dosing group	At 12 months, N=4 (out of N=5) in the DR group vs. N=1 in the standard dosing group with a persistent flare had not regained PASI and DLQI ≤5	Successful DR: N=28 (53%) (N=16 ADA, N=3 ETN, N=9 UST). N=10 were taking 67% of the original dose and N=18 were taking 50% of the original dose.<br>DR vs. standard dosing group: Median PASI was 3.4 vs. 2.1. Median DLQI was 1.0 vs.0. Short flares: 36% vs. 14%. Persistent flare: 9% vs. 9%.
**Baniandrés et al., 2015 [28]**	Retrospective cohort, observational (uncontrolled; UC)	-	ADA, ETN, IFX, UST	104 (56 DR, 48 standard dosing)	≥6 months	PASI90 for ≥6 months	Stepwise prolongation to:<br>ADA Q3W-Q4W-Q6W<br>ETN 25mg QW- Q10D or 50mg Q10D-Q2W<br>IFX Q9W-Q11W	Retreatment with standard dose if: loss of PASI90-100 response	-	-	All patients on DR achieved and maintained ≥PASI75.

							UST 45mg Q13W-Q14W				
**Bardazzi et al., 2016 [30]**	Retrospective cohort, observational (UC)	-	IFX	20 (all DR)	≥12 months	PASI 0 for ≥12 months	Fixed interval: 5mg/kg Q10W	Retreatment with standard dose if: loss of PASI50 response	-	-	N=5 (25%) of the patients on DR experienced a relapse.
**van Bezooijen et al., 2017 [27]**	Prospective cohort, observational (UC)	72 weeks (after 6 weeks run-in period)		42 (all DR)[c]	6 weeks run-in period	PASI <8 and a run-in period of 6 weeks before study initiation was used, in which the PASI score was not allowed to fluctuate ≥3 points	Stepwise prolongation every 12 weeks to:<br>ADA Q3W-Q4W<br>ETN QW-Q2W<br>UST 45mg:<br>45mg Q16W<br>45mg Q20W<br>45mg Q24W<br>UST 90mg:<br>90mg Q12W<br>45mg Q12W	Retreatment with previous effective dose if:<br>An unacceptable increase in disease activity, judged by the patient or PASI score >8	-	-	Successful interval prolongation at week 78: ADA: 1/16 (Q3W), 6/16 patients (Q4W). Failures N=6. Lost to follow-up N=3.<br>ETN: 5/16 (Q2W). Failures N=9. Lost to follow-up N=1. Consent withdrawn N=1.<br>UST 45mg: 2/9 (Q24W). Failures N=7.<br>UST 90mg: failure N=1.<br>Successful interval prolongation was defined as PASI ≤ 8 and without loss of self-reported efficacy.
**Blauvelt et al., 2017 [37]**	Randomized controlled trial, double-blind PSTELLAR	± 108 weeks (after 16 weeks induction phase)[d]	UST	378 (196 DR, 84 failed DR, 76 standard dosing and 22 lost to follow-up)	28 weeks	PGA 0/1 at week 28	Based on dose interval determination period[d]: UST Q12W-Q16W-Q20W- Q24W	-	-	-	70% (N=196) of the patients on DR and 70% (N=53) of the standard dosing patients maintained a PASI75 response at all seven assessments period visits.
**Carrascosa et al., 2015 [39]**	Retrospective cohort, observational (UC)	-	ADA, ETN, IFX, UST	637 (223 DR, 368 standard dosing)[e]	-	-	Dose adjustment: ADA, ETN,	-	-	-	DR vs. standard dosing: mean PASI of 1 (N=140) vs. mean PASI of 2.6 (N=231) at the cut-off date (Jan 2014).

	BIOBADADERM						UST and IFX (not specified)[f]				
**Esposito et al., 2017 [40]**	Retrospective cohort, observational (UC)<br>ONDA	-	ADA, ETN, IFX (no UST DR)	350 (47 DR of which 27 due to persistent remission)[g]	-	"Remission"	Dose adjustment: ADA, ETN, IFX (not specified)	-	-	-	PASI75 response rate at week 96 was higher in the DR group as compared to the standard dosing group (not significant).
**Fotiadou et al., 2012 [31]**	Retrospective cohort, observational (UC)	-	ADA	52 (14 DR)	≥ 12 months	PASI100 response after 12 months[h]	Fixed interval: ADA Q3W	-	No relapse was observed	NA	All patients on DR sustained PASI100 response, with 10 patients (71%) having completed the 30 months of follow-up (since baseline).
**Hansel et al., 2017 [32]**	Retrospective cohort, observational (UC)	-	ADA	30 (all DR)	≥ 12 months	Sustained PASI100 response for ≥ 12 months	Stepwise prolongation: With 3-4 days every month to ADA Q3W-Q4W	Retreatment with standard dose if: ≥20% loss of PASI response	Mean time to relapse (N=12) was 3.8 months (SD 0.9 months) (median time to relapse was 3 months (95%CI 1.9–4.1))	All patients who relapsed (N=12) achieved PASI100 ± 1 month after retreatment with the standard dose	18 of the patients on DR (60%) maintained PASI100 (3 patients using ADA Q3W and 15 patients with ADA Q4W). Median observation period was 60 months (min. 36, max. 66 months).

**Lebwohl et al., 2015 [26]**	Randomized controlled trial, double-blind (Phase III) AMAGINE-2, AMAGINE-3	40 weeks (after a 12 week induction phase)	BRO	2374 (1698 DR, 676 standard dosing)	12 weeks	-	Fixed interval: 140 mg Q2W (N=680) or 140 mg Q4W (N=676) or 140 mg Q8W (N=342) or 210 mg Q2W (N= 676)[i]	Retreatment with 210 mg Q2W if: single sPGA score ≥3 or persistent sPGA scores of 2 over ≥4-week period at week 16	-	-	Maintenance of sPGA 0/1 at week 52 - DR vs. standard dosing:<br>AMAGINE-2: 140 mg Q2W: N= 144 ((43%); 95%CI 37-48); 140 mg Q4W: N=30 ((9%); 95%CI 6-13);<br>140 mg Q8W: N=8 ((5%); 95%CI 2-9) vs. 210 mg Q2W: N=209 ((63%), 95%CI 57-68).<br>AMAGINE-3: 140 mg Q2W: N=154 ((45%); 95%CI 40-50); 140 mg Q4W: N=53 ((16%); 95%CI 12-20);<br>140 mg Q8W: N=10 ((6%); 95%CI 3-10) vs. 210 mg Q2W: N=208 ((61%); 95%CI 55-66).<br>In both trials 210 mg Q2W showed a significant difference in effect compared to 140 mg Q2W/Q4W/Q8W.
**Lee et al., 2018 [35]**	Retrospective cohort, observational (UC)	-	ADA	62 (22 DR of which 10 due to "well controlled disease")	-	Prolongation due to "well controlled disease" (not adamant for DR)	Dose adjustment: ADA > Q2W, up to Q4W (not specified)	-	-	-	8/9 (89%) of the patients on DR who were in remission maintained "good disease control" for ≥ 6 months after DR. 1 of 9 patients was lost to follow-up. One patient had a dose reduction due to mild disease and an adverse event.
**López-Ferrer et al., 2013 [33]**	Retrospective cohort, observational (UC)	-	ADA	119 (32 DR)	-	PASI75 response within the first 16 weeks of treatment	Dose adjustment: ADA Q3W-Q4W (not specified) for	Retreatment with previous effective dose if: partial loss of response occurred (not specified)	-	-	Treatment retention rate was 31/32 (97%) in patients with a prolonged interval. 3/32 (9.4%) of the patients on DR reinstalled their previous effective dose due to partial loss of response.

						and PASI <5 persistently thereafter	variable periods of time				
**Ovejero-Benito et al., 2020 [41]**	Prospective cohort, observational (UC)	-	ADA, ETN, UST	120 (183 treatment cycles, DR was applied in 59 cycles and standard dosing in 124 cycles)	2 consecutive visits (not specified)	PASI90 or PASI <3 at two consecutive visits	-	-	-	-	Successful DR was achieved: 96% (26/27) in ADA, 100% (3/3) in ETN and 76% (22/29) in UST patients (183 drug cycles in total).DR was considered successful if PASI90 or < 3 was maintained for ≥ 6 months.
**Piaserico et al., 2016 [34]**	Retrospective cohort, observational (UC)	≥ 12 months	ADA, ETN	85 (all DR)	≥ 12 months	PASI 0 ≥ 12 months	Fixed interval: ADA Q3W ETN Q10D	Retreatment with standard dose if: ≥ 50% loss of PASI improvement	Mean time to relapse was 48 months (CI95% 43–52.7) for ADA and 39.3 months (CI95% 33.7–44.8) for ETN. The cumulative relapse risk was 0%, 12% and 20% for ADA and 14%, 31% and 39% for ETN after, respectively, 3, 6 and 12 months	All patients that experienced relapse and were treated with the standard dose showed a rapid response (not specified)	-

**Puig et al., 2013 [42]**	Retrospective cohort, observational (UC)	-	ADA, ETN, IFX, UST (no IFX and UST DR)	78 (9 DR)	-	-	Dose adjustment: ADA, ETN (not specified)	-	-	-	Based on the inclusion criteria of the study, we assumed that the DR patients must have gained at least ≥ PASI75 response and PASI <5.
**Reich et al., 2020 [38]**	Randomized controlled trial, open-label, rater-blinded (phase IIIb)	28 weeks (after 24 weeks induction phase)	SEC	1306 (662 DR, 644 standard dosing)	≥ 6 months	≥ PASI90	Fixed interval: SEC 300mg Q6W	-	-	-	At week 52, 85.7% of patients in the standard dosing group (Q4W) vs. 74.9% of patients in the DR group (Q6W) maintained PASI90 response (OR 1.91, 95%CI 1.44–2.55). Both groups (Q4W vs. Q6W) did not markedly differ in their PASI50 (99.7% vs. 99.2%) and PASI75 (97.9% vs. 93.5%) responses at week 52.
**Romero-Jimenez et al., 2016 [29]**	Retrospective cohort, observational (UC)	≤ 12 months	ADA, ETN, IFX, UST	224 (118 in the post-protocolization group, in which 39 DR)[j]	≥ 6 months	PASI90–100 ≥ 6 months	Stepwise prolongation:<br>ADA Q3W<br>ETN Q10D<br>IFX Q9W<br>UST Q13W.<br>Intervals could be further prolonged in the same manner if remission continued for another 6 months	Retreatment with previous effective dose if: loss of PASI90-100	-	-	The dose was lower in 43.4% of ADA patients (N=23), 37.5% of ETN patients (N=9), 28.6% of INF patients (N=2) and 14.7% of UST patients (N=5).

**Romero-Jimenez et al., 2018 [43]**	Retrospective cohort, observational (UC)	-	UST	62 (14 DR, 34 standard dosing)[k]	Median (p25;p75) time to DR was 22.6 months (10.0;37.0)	-	Dose adjustment: UST (not specified)	-	-	-	-
**Taniguchi et al., 2013 [36]**	Prospective cohort, observational (UC)	36 weeks (after 24 weeks induction phase)	ADA	17 (10 DR, 7 standard dosing)	≥ 6 months	"Patients' preferences"	Fixed interval: ADA Q4W				At week 60, DR vs. standard dosing: PASI50: 90% vs.100%; PASI75 90% vs. 85.7%; PASI90 40% vs.57.1%. In both groups, all patients who achieved PASI75 at week 24 maintained PASI75 response at week 60. Furthermore, PASI50 and PASI90 response rate at week 60 were also comparable between both groups (P=1 and P=0.63).

ADA = adalimumab (standard dose 40 mg Q2W); ETN = etanercept (standard dose 50 mg QW); IFX = infliximab (standard dose 5 mg/kg Q8W); UST= ustekinumab (standard dose 45 mg Q12W (≤100 kg) or 90 mg Q12W (>100kg); SEC = secukinumab (standard dose 300 mg Q4W); BRO = brodalumab (standard dose 210 mg Q2W); GUS = guselkumab (standard dose 100mg Q8W); mg = milligram, Q = every, W = weeks, D = days; e.g. Q2W meant every 2 weeks; DLQI = Dermatology Life Quality Index; PGA = Physician Global Assessment; PASI = Psoriasis Area and Severity Index; PASI50/75/90/100 = 50/75/90/100% improvement in Psoriasis Area and Severity Index; sPGA = static Physician Global Assessment; 95%CI = 95% confidence interval; SD = standard deviation; UC = uncontrolled.
[a]Follow-up post-tapering; [b]Number of patients included per-protocol; [c]In this review only the patients in the study of van Bezooijen et al. who tapered 'per-label' were included; [d]Blauvelt et al. based the extension of the dosing interval on a dose-interval determination period after 16 weeks of open-label treatment with ustekinumab. From week 28, patients did not receive ustekinumab until the next visit (scheduled every 4 weeks) at which PGA 0/1 was not maintained. Patients who failed to maintain PGA 0/1 at 16, 20 or 24 weeks after their last injected dose continued to receive ustekinumab according to their last effective dose interval (last visit were PGA 0/1 was maintained). The total follow-up was 124 weeks (28 week open-label run in period, follow-up by 96 week double-blinded treatment and follow-up period); [e]Carrascosa et al. included 637 patients of which 368 received standard dosing 223 patients reduced their dose and 46 patients escalated their dose; [f]Carrascosa et

al. reported that 100% of the patients on adalimumab (N=95) and ustekinumab (N=71) and 79% of the patients on etanercept (N=33) extended the dosing interval. 73% (N=11) of patients on infliximab decreased the dose per gift; [g]In the study by Esposito et al. 27 of the 47 patients tapered due to persistent remission (10/14 patients on adalimumab, 13/22 on etanercept and 4/11 on infliximab). Numbers were confirmed in correspondence with the author; [h]Fotiadou et al. reported that the dose interval was increased in patients who "who achieved and maintained a PASI100" after the first year of treatment; [I]In the study by Lebowhl et al., the number of patients combined for AMAGINE-2 and 3 was: AMAGINE-2, according to dose regimen: 140 mg Q8W: N=168; 140 mg Q4W: N=335; 140 mg Q2W: N=337; 210 mg Q2W: N= 334. AMAGINE-3: 140 mg Q8W: N=174; 140 mg Q4W: N=341; 140 mg Q2W: N=343; 210 mg Q2W: N=342; [j]Romero-Jimenez 2016 et al. assessed the impact of a protocol on dose optimization of biologic therapy, in this review only the post- protocol group is included; [k]Romero-Jimenez 2018 et al. included 62 patients of which 34 received standard dosing, 14 patients reduced their dose and 14 patients escalated their dose.
