# Supplementary material for: Dose reduction of biologics in patients with plaque psoriasis: a review
Source: Front Pharmacol. 2024 Mar 28;15:1369805. doi: 10.3389/fphar.2024.1369805 (PMC11007084; doi:10.3389/fphar.2024.1369805)
Supplement: Supplementary file 4 [file DataSheet1.PDF]

Supplementary appendix 3: Detailed description of studies on effectiveness of dose reduction

Atalay et al., – prospective cohort (N=88) (1-year extension study of randomized CONDOR trial) on adalimumab, etanercept, ustekinumab

In the 1-year extension study of the CONDOR trial a subcohort of a total of 88 patients was followed for another year after the end of the trial, resulting in a total follow-up of two years of this specific cohort.(Atalay et al., 2022) The subcohort comprised patients from one center who were initially randomized to a reduced dose (N=44/88) or standard dose (usual care, UC) (N=44/88) of adalimumab (DR N=18; UC N=17), etanercept (DR N=11; UC N=12) or ustekinumab (DR N=15; UC N=15) at the start of the CONDOR trial. In the trial, DR was performed stepwise by interval prolongation of firstly 67% of standard dose and secondly 50% (adalimumab 40mg Q3W/Q4W, etanercept 50mg Q10D/Q2W, ustekinumab 45mg/90mg Q18W/Q24W).(14) DR was only applied when patients had an absolute PASI and DLQI ≤5 for ≥6 months. In case of relapse after DR (PASI and/or DLQI >5), retreatment with the previous effective dose was applied. Effectiveness, or success, of DR was shown as the percentage of patients that maintained an absolute PASI (and DLQI) score ≤5 during the extension phase. Results were not specified per biologic but on a total study population level. At the end of the 1-year CONDOR trial, 59% of the patients initially randomized to DR (26/44 patients) was still on a low dose. At the end of the 1-year extension study (i.e. 2 years after CONDOR initiation), 69% of this group (18/26 patients) was still on a low dose. Of these 18 patients on a lower dose at end of the extension period, 7/18 patients (39%) used 67% of the standard dose and 11/18 patients (61%) used 50% of the standard dose. Over the total two years of follow-up (i.e. 1-year CONDOR and 1-year extension), a total of 10 patients relapsed after DR of which 80% (8/10 patients) regained PASI and/or DLQI <5 after retreatment with the previous effective dose within the observation period.(Atalay et al., 2022)

Atalay et al., - prospective cohort (N=80) (one-step DR strategy) on adalimumab, etanercept, ustekinumab

In this prospective cohort study, a total of 80 patients who started with a one-step DR strategy of adalimumab (N=42), etanercept (N=16) or ustekinumab (N=22), were followed for on average one year after DR start (total follow-up duration ranged from 49 to 670 days).(Atalay et al., 2021) DR was performed by fixed interval prolongation in one DR step, lowering the dose to 67% of standard dose (adalimumab 40mg Q3W, etanercept 50mg Q10D, ustekinumab 45mg Q18W). DR was only applied when patients had absolute PASI and DLQI ≤5 for ≥6 months. In case of relapse after DR (PASI and/or DLQI >5), retreatment with standard dose or previous effective dose was applied. Effectiveness of DR was shown as the percentage of patients that discontinued DR, both of the total study population and the population per biologic. Of the total study population, 45% (36/80 patients) discontinued DR. The overall median time to stop DR was 19 months (95% CI 14.9-23.1). Reasons for discontinuing DR was reduced effectiveness as reported by patients (N=18/36; 50%) or by both patient and physician (N=9/36; 25%). Of the patients who reduced adalimumab to 40mg Q3W, 45% (19/42 patients) discontinued DR. Median time to stop DR was 9 months (95% CI 14.7-23.3). Of the patients who reduced etanercept to 50mg Q10D, 44% (7/16 patients) discontinued DR. After 21 months (±630 days), at end of analysis, >50% of patients was still active on DR in the survival curve, therefore a median time to stop DR could not be calculated. Of the patients who reduced ustekinumab to 45mg/90mg Q18W, 46% (10/22 patients) discontinued DR. Median time to stop DR was 19 months (95% CI 12.0-26.0). Results on the effectiveness of

retreatment after relapse were shown only on total study population level. Over the total follow-up period, a total of 8 out of 80 patients (10%) relapsed after DR, of which 50% (N=4) continued DR at own request and 50% (N=4) returned to standard dose. A PASI <5 was regained within 6 months for 100% of patients that continued DR and for 75% of patients that returned to standard dose (3/4 patients).(Atalay et al., 2021)

Di Altebrando et al., – prospective cohort (N=199) on adalimumab, etanercept, infliximab, ustekinumab

In this prospective cohort study, a total of 199 patients of which 96 patients started with DR and 103 patients continued standard dose (UC) of adalimumab (DR N=47; UC N=34), etanercept (DR N=16; UC N=25), infliximab (DR N=21; UC N=7) or ustekinumab (DR N=12; UC N=37), were followed for a maximum of ±102 months after start DR.(Di Altobrando et al., 2022) DR was performed by fixed interval prolongation, lowering the dose of adalimumab to 40mg Q3W (67% of standard dose), etanercept to 50mg Q10D (67%), infliximab to 5mg/kg Q10W (80%), and ustekinumab to 45mg/90mg Q14W (86%). DR was only applied when patients had a relative PASI 75-100 for ≥1 year. In case of relapse after DR (≥50% worsening of initial PASI 75-100), retreatment with standard dose was applied. Effectiveness of DR was shown as the percentage of patients with relapses and presented per biologic. Also results on effectiveness of retreatment after relapse were shown per biologic. During follow-up, a total of 26 out of 96 patients (27%) on DR relapsed. Of the patients who reduced adalimumab to 40mg Q3W, 36% (17/47 patients) relapsed after on average 10 months. Of the patients who reduced etanercept to 50mg Q10D, 6% (1/16 patients) relapsed after 27 months. Of the patients who reduced infliximab to 5mg/kg Q10W, 24% (5/21 patients) relapsed after on average 31 months. Of the patients who reduced ustekinumab to 45mg/90mg Q14W, 25% (3/12 patients) relapsed after a mean time of 17 months. Of all 26 relapsed patients, 96% (25/26 patients) regained their initial PASI after retreatment with standard dose.(Di Altobrando et al., 2022)

Herranz-Pinto et al., – retrospective cohort (N=69) on guselkumab

This retrospective cohort study included a total of 69 patients of which 45 patients underwent an ‘on-demand’ DR strategy of guselkumab.(Herranz-Pinto et al., 2023) After an initial complete response to guselkumab after the first three administrations (at weeks 0, 4 and 12), patients re-administered guselkumab only when absolute PASI reached ≥1. Note that 36% of the patients did not follow the complete induction scheme. Follow-up visits took place between predefined time intervals reflecting interval duration between doses over a period of 88 weeks. Patients were divided into four groups: one standard dose group and three groups based on the % DR of the standard dose. The “blue group” had a reduction of >20% <40% (N=24), the “orange group” >40% <60% (N=10), and the “red group” >60% (N=11). Effectiveness of DR was defined by comparing absolute PASI scores at multiple time points (11-90 weeks) with baseline. An average reduction of 29% (guselkumab 100mg Q11W), 52% (guselkumab 100mg Q17W) and 71% (guselkumab 100mg Q27W) was reached in the blue, orange and red group, respectively. All DR groups showed a significant decrease in PASI between weeks 11-20 compared to baseline. See S2 for a more extensive overview. A retreatment strategy in case of relapse after DR was not specified. Drug survival curves for discontinuation due to an adverse event, lack of efficacy, or death were analyzed. After 1 year, the curves showed a survival rate of 93.5% in the overall population (including patients on standard dose), of 94.4% in the blue group, and 100% in the orange and red group without significant differences between groups (p=0.48).(Herranz-Pinto et al., 2023)

Van der Schoot et al., – prospective cohort study (N=59) on effectiveness of retreatment of adalimumab, etanercept, and ustekinumab

One prospective cohort study of van der Schoot et al., specifically analyzed effectiveness of retreatment with standard dose in case of relapse after DR in patients using adalimumab (N=23), etanercept (N=16) or ustekinumab (N=20).(van der Schoot et al., 2022) In this cohort study, a total of 59 patients who returned to standard dose after DR and who were originally included in the previously mentioned studies on DR strategies (randomized CONDOR trial and one-step DR cohort study) were prospectively followed for 2 years after returning to standard dose.(Atalay et al., 2020, Atalay et al., 2021, van der Schoot et al., 2022) Results on effectiveness of retreatment with the standard dose after relapse was shown on total study population level. A total of 40 out of 59 patients (68%) returned to the standard dose based on protocol (PASI and/or DLQI >5) and 19/59 patients (32%) at their own request. After 1 year of retreatment with the standard dose, absolute PASI was comparable to PASI at start of DR; median [interquartile range (IQR)] PASI 2.4 [1.5-3.0] (median difference [IQR] 0.0 [-0.8-1.5]).(van der Schoot et al., 2022)

References in supplemental 3

ATALAY, S., VAN DEN REEK, J., DEN BROEDER, A. A., VAN VUGT, L. J., OTERO, M. E., NJOO, M. D., MOMMERS, J. M., OSSENKOPPELE, P. M., KOETSIER, M. I., BERENDS, M. A., VAN DE KERKHOF, P. C. M., GROENEWOUD, H. M. M., KIEVIT, W. & DE JONG, E. 2020. Comparison of Tightly Controlled Dose Reduction of Biologics With Usual Care for Patients With Psoriasis: A Randomized Clinical Trial. *JAMA Dermatol,* 156**,** 393-400.

ATALAY, S., VAN DEN REEK, J., GROENEWOUD, J. M. M., VAN DE KERKHOF, P. C. M., KIEVIT, W. & DE JONG, E. 2022. Two-year follow-up of a dose reduction strategy trial of biologics adalimumab, etanercept, and ustekinumab in psoriasis patients in daily practice. *J Dermatolog Treat,* 33**,** 1591-1597.

ATALAY, S., VAN DER SCHOOT, L., VANDERMAESEN, L., VAN VUGT, L., EILANDER, M., VAN DEN REEK, J. & DE JONG, E. 2021. Evaluation of a One-step Dose Reduction Strategy of Adalimumab, Etanercept and Ustekinumab in Patients with Psoriasis in Daily Practice. *Acta dermato-venereologica,* 101**,** adv00463.

DI ALTOBRANDO, A., MAGNANO, M., OFFIDANI, A., PARODI, A., PATRIZI, A., CAMPANATI, A., BURLANDO, M. & BARDAZZI, F. 2022. Deferred time of delivery of biologic therapies in patients with stabilized psoriasis leads to a 'perceived satisfaction': a multicentric study. *J Dermatolog Treat,* 33**,** 415-419.

HERRANZ-PINTO, P., ALONSO-PACHECO, M., FELTES-OCHOA, R., MAYOR-IBARGUREN, A., SERVERA-NEGRE, G., BUSTO-LEIS, J., GONZALEZ-FERNÁNDEZ, M. & HERRERO-AMBROSIO, A. 2023. Real-world Performance of a New Strategy for Off-Label Use of Guselkumab in Moderate to Severe Psoriasis: Super-Responder Patients as the Epitome of Efficacy and Optimisation. *Clinical drug investigation*.

VAN DER SCHOOT, L., ATALAY, S., OTERO, M., KIEVIT, W., VAN DEN REEK, J. & DE JONG, E. 2022. *Regaining adequate treatment responses in patients with psoriasis who discontinued dose reduction of adalimumab, etanercept or ustekinumab,* England.
